# Effects of LinTT1-peptide conjugation on the properties of poly(ethylene glycol)-block-(ε-caprolactone) nanoparticles prepared by the nanoprecipitation method

**DOI:** 10.1007/s13346-024-01768-7

**Published:** 2025-01-03

**Authors:** Voitto Känkänen, Sami-Pekka Hirvonen, Tambet Teesalu, Jouni Hirvonen, Vimalkumar Balasubramanian, Hélder A. Santos

**Affiliations:** 1 Drug Research Program, Division of Pharmaceutical Chemistry and Technology, Faculty of Pharmacy, University of Helsinki, Helsinki, FI-00014 Finland; 2 Department of Applied Physics, School of Science, Aalto University, FI-02150 Espoo, Finland; 3 Department of Chemistry, Faculty of Science, University of Helsinki, P. O. Box 55, Helsinki, 00014 Finland; 4 Laboratory of Precision- and Nanomedicine, Institute of Biomedicine and Translational Medicine, University of Tartu, Tartu, 50411 Estonia; 5 Materials Research Laboratory, University of California, Santa Barbara, CA 93106 USA; 6 Advanced Drug Delivery, Bayer Oy, Turku, FI-20210 Finland; 7 Department of Biomaterials and Biomedical Technology, The Personalized Medicine Research Institute (PRECISION), University Medical Center Groningen, University of Groningen, Ant. Deusinglaan 1, Groningen, 9713 AV The Netherlands

**Keywords:** Block copolymers, Peptides, Nanoparticles, Nanoprecipitation, Conjugation, Targeting

## Abstract

Functionalization of polymer nanoparticles (NPs) with targeting peptides is of interest for drug delivery applications to enhance tumor accumulation and penetration. Herein, we evaluated the feasibility of two different methods for the attachment of a tumor-penetrating peptide LinTT1 (AKRGARSTA) to poly(ethylene glycol)-block-poly(ε-caprolactone) (PCL-PEG) NPs: (1) “post-conjugation” onto pre-formed nanoparticles, and (2) “pre-conjugation”, the synthesis and purification of peptide-polymer conjugates and subsequent nanoprecipitation of the conjugates diluted with non-functionalized polymers. Conjugation of the labelled peptide via maleimide-thiol chemistry was verified by gel permeation chromatography (GPC) and fluorescence measurements. Characterization of NPs with respect to particle size, zeta potential, morphology and peptide content was performed, and their ability to bind to the target protein p32 was tested using a cell-free assay. Importantly, both methods resulted in NPs that were able to bind their target when methyl-terminated PCL-PEG used as the diluent polymer, but not when acid-terminated polymer was used. Moreover, peptide conjugation induced a morphological transformation from spheres to vesicles regardless of the conjugation method used. However, smaller and more homogeneous NPs were obtained by the pre-conjugation method.

**Supplementary Information:**

The online version contains supplementary material available at 10.1007/s13346-024-01768-7.

## Introduction

Targeted drug delivery using NPs functionalized with biomolecules has been a subject of active research in the recent decades, particularly using peptide-based targeting ligands [1–3]. Short peptides have several favorable properties as targeting ligands, including low cost and ease of preparation with high purity by solid-phase synthesis [4]. For covalent conjugation of peptides to NPs, maleimide-thiol Michael addition reaction is one of the preferred methods due to high reaction rate and specificity to thiols at neutral pH, which allows selective conjugation via cysteines [5–7].

The most common approach in the literature appears to be conjugation of peptide onto NPs after particle formation (*post-conjugation*). This method is generally favored, because it is thought to ensure integrity of the peptide and its optimal presentation on the NP surface [8]. If the NP manufacturing process is hostile to the peptide structure, post-conjugation is obviously the only feasible method. However, for amphiphilic block copolymer (ABC) NPs that can be formed by macromolecular self-assembly under mild conditions, a *pre-conjugation* approach has also been shown to be feasible [8–10]. In the pre-conjugation approach, NPs are prepared from peptide-polymer conjugates that self-assemble [11] in aqueous environments to form NPs, and this is assumed to lead to peptide presentation on NP corona based on hydrophilic nature of the peptide [8]. However, the conjugation of ligands can alter the copolymer self-assembly behavior, which can in turn affect the formation of NPs and potentially cause the ligand to be trapped inside the nanoparticle structure, unavailable for binding to its target receptor [8].

We expect the pre-conjugation approach to have several benefits in drug delivery system manufacturing. Firstly, it avoids any risk of cross-reaction of maleimides with free thiols or amines in the active pharmaceutical ingredient, which can be an issue with formulations of protein or peptide drugs. Secondly, a major limitation of the maleimide-thiol reaction is the possibility of ligand switching or *payload migration* by retro-Michael addition, and to prevent this, additional post-processing steps are required to hydrolyze the maleimide ring structure [12]. With the pre-conjugation approach, this lengthy hydrolysis step can be performed and verified before introducing the material in the final manufacturing process. Thirdly, with the use of pre-synthesized, purified, and well-characterized peptide-polymer conjugates, any uncertainties related to the conjugation reaction step or the presence of unreacted peptide in final drug product manufacturing can be avoided [8]. Reduction of post-processing steps for drug-loaded particles also alleviates possible concerns with drug leakage from NPs and colloidal or structural instability of the drug delivery systems during these steps [13].

Controlling the surface chemistry of NPs is of utmost importance, as it largely determines the NPs’ interactions with cells and tissues, as well as the physical properties, such as charge and colloidal stability and processability [14, 15]. GPC is a size-based separation method routinely used for molecular weight distribution (MWD) analysis of polymers. However, this method has also been applied to the analysis of polymer-biomolecule conjugates, as multi-detector GPC can enable simultaneous analysis of composition and MWD [16–19].

For the self-assembly of diblock copolymers, volume fraction of the hydrophilic block, $\:f$, is an important parameter that determines the thermodynamically favored morphology of polymer aggregates in solution [11]. PEG-PCL polymers with relatively long PCL blocks (*f* ≤ 0.25) were shown to preferentially form worm-like micelles or bilayer vesicles in aqueous solution [20]. However, PCL-PEG with low *f* values were also used to prepare solid spherical NPs through control of particle formation kinetics, by adjusting nanoprecipitation process parameters, such as solvent and temperature [21–23]. In previous work [24, 25], we showed that PCL-PEG polymers with $\:f$ ≈ 0.16 (10–12 kDa PCL and 2 kDa PEG) can be used to prepare solid spherical nanoparticles by the nanoprecipitation method, and that these NPs can be loaded with high amounts of a hydrophobic model drug.

In this work, we sought to prepare peptide-conjugated solid spherical nanoparticles from these low $\:f$ diblock copolymers. We explored the pre- and post-conjugation approaches for the attachment of LinTT1, a short linear cationic peptide, LinTT1 (AKRGARSTA, Ala-Lys-Arg-Gly-Ala-Arg-Ser-Thr-Ala) by reacting a terminal cysteine residue of a modified version of the peptide with maleimide-terminated PCL-PEG. LinTT1 is a tumor-penetrating peptide, and its primary molecular target is the p32 protein (also known as gC1qR) that is overexpressed on various tumors and the associated vasculature [26–28]. We first prepared LinTT1-conjugated NPs by the post-conjugation method and evaluated the effects of conjugation on particle size and colloidal stability. We then explored the pre-conjugation method by synthesizing and purifying polymer-peptide conjugates, characterizing them using GPC methods and spectrophotometry and preparing NPs from the conjugates. We characterized the NPs by light scattering techniques, electron microscopy and fluorescence analysis. Finally, the ability of the NPs to bind to their target protein p32 was evaluated in vitro using a cell-free assay.

## Materials and methods

### Materials

**Fig. 1:**
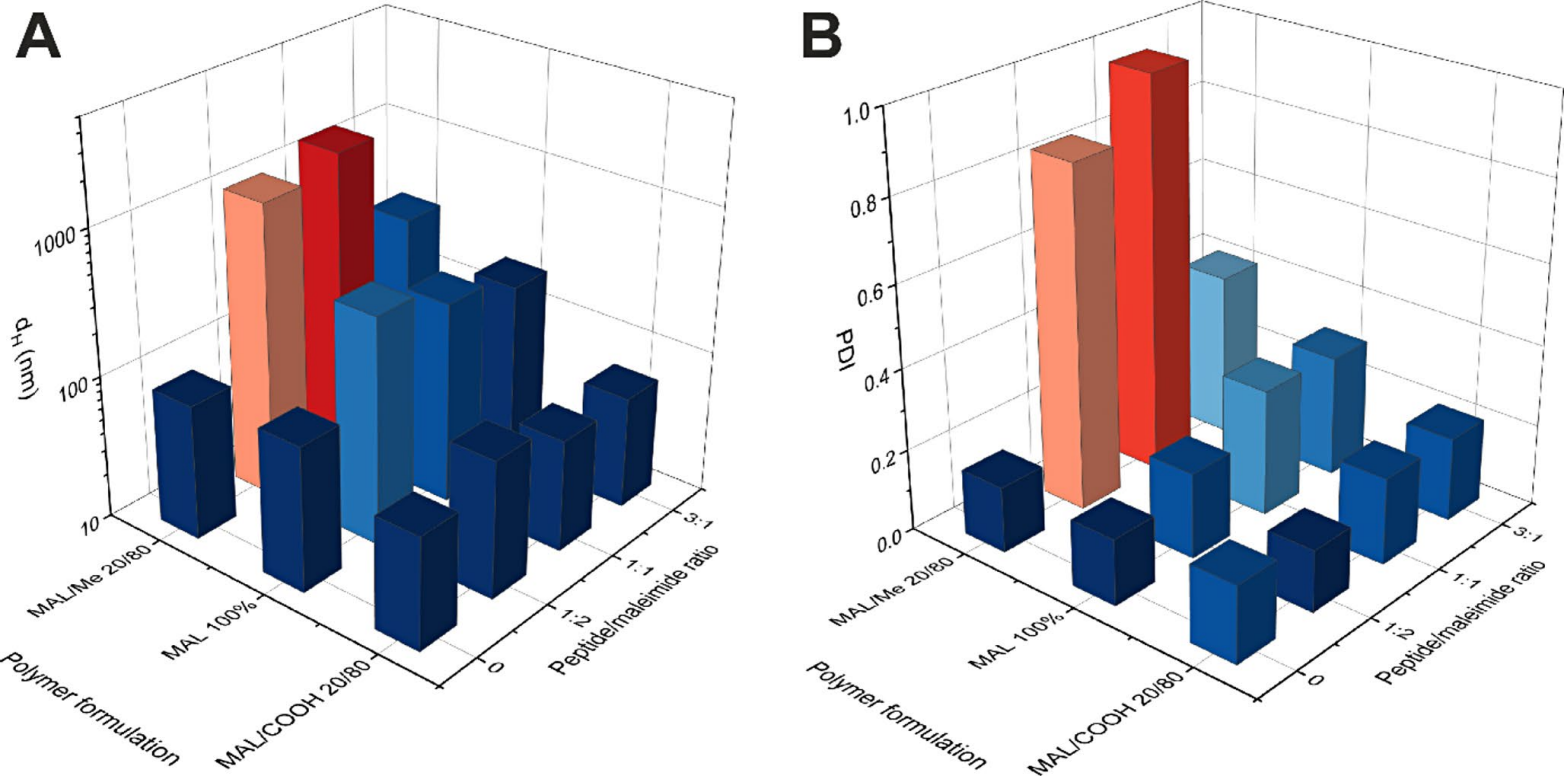
Peptide conjugation in salt-free 25 mM HEPES (pH 7.0) buffer affected the PDI (**A**) and particle size d_H_ (**B**) of the NPs, depending on the polymer mixture used

**Fig. 2:**
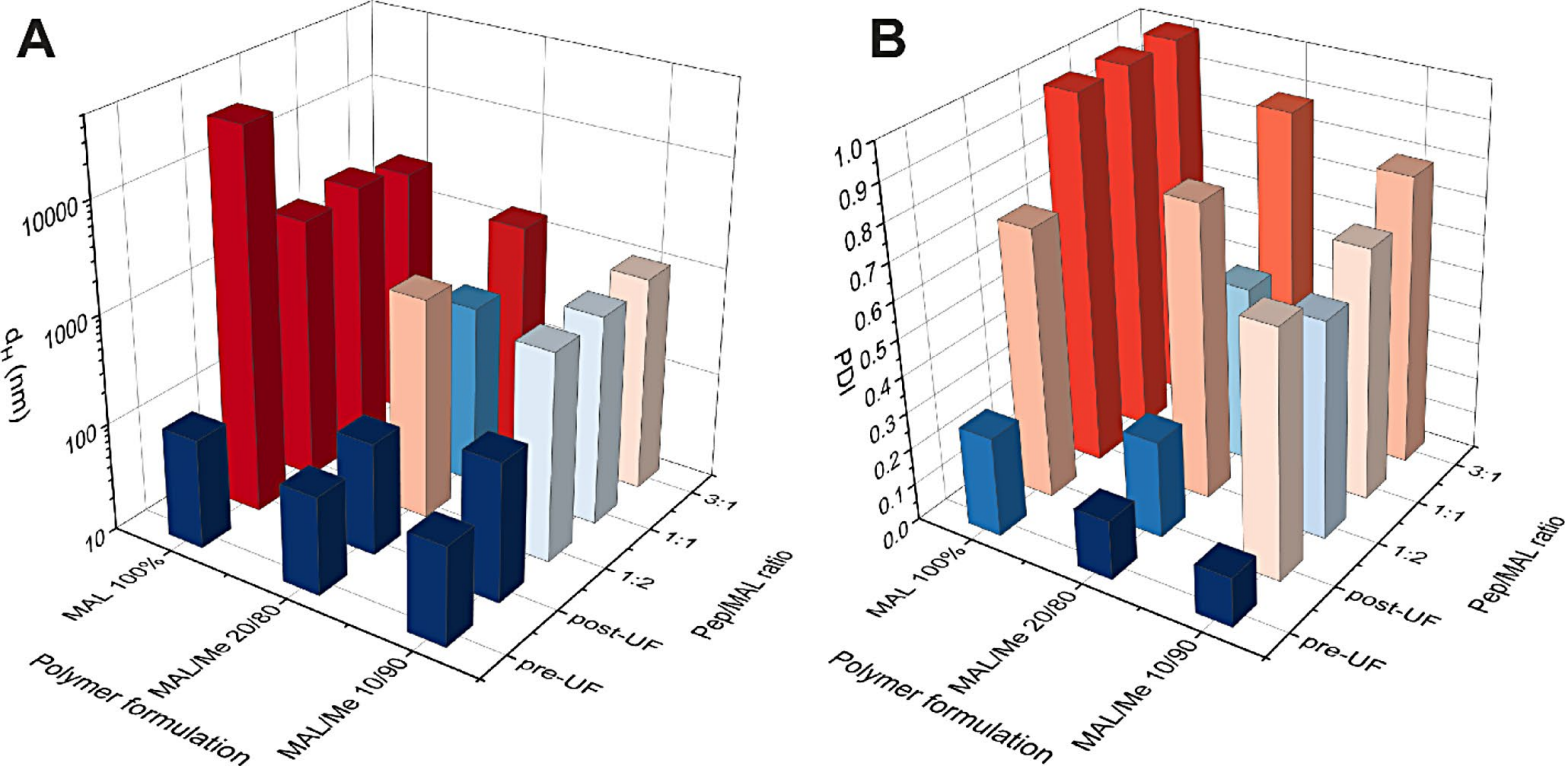
Peptide conjugation in phosphate buffer with 150 mM NaCl (pH 7.4) caused an increase in average d_H_ (**A**) and PDI (**B**) of NPs composed of mixture of methyl- and maleimide-terminated polymers

Poly(ethylene glycol)-block-(ε-caprolactone) diblock copolymers (PCL-PEG) with different PEG termini were purchased from various suppliers: PCL-PEG-Me (nominal mol. wt. 10k-2k and 6k-2k) were purchased from PolymerSource Inc., Canada. PCL-PEG-COOH (nominal mol. wt. 12k-2k) was purchased from Akina Inc., IN, USA. For the initial post-conjugation tests (Figs. 1 and 2), PCL-PEG-MAL with nominal mol. wt. 10k-2k from CreativePEGWorks, NC, USA was used. For colloidal stability experiments shown in Table S1−S5 and Figures S1−S8, PCL-PEG-MAL with nominal mol. wt. 10k-2k from Akina Inc. was used. For all other experiments, in-house synthesized PCL-PEG-MAL was used. The control fluorophore 5-FAM-Cys and the fluorescently labelled LinTT1 peptides 5-FAM-Cys-Ahx-AKRGARSTA-OH and 5-FAM-Cys-Ahx-AKRGARSTA-NH_2_ were purchased from TAG Copenhagen A/S (Denmark). Ni-NTA magnetic agarose beads (Ref. 36113) were purchased from Qiagen. 6xHis-tagged p32 was prepared in-house as described earlier [27].

Phosphate-buffered saline concentrate (10xPBS, 67 mM sodium phosphate, 1.5 M NaCl) was from Cytiva. Poloxamer (Kolliphor P188 micro Geismar) was purchased form BASF. Imidazole (≥ 99.5%), Bovine Serum Albumin (BSA, ≥ 96%), HEPES (4-(2-hydroxyethyl)-1-piperazineethanesulfonic acid, ≥ 99.5%) and acetone (≥ 99.5%) were obtained from Sigma. Deionized water was obtained with a Milli-Q^®^ Integral 15 Water Purification System with a Millipak^®^ Express 40 filter (Merck Millipore). TCEP hydrochloride (≥ 98.0%) was obtained from TCI Chemicals. For the syntheses, anhydrous DCM and DMF obtained Sigma Aldrich were used. Methanol (≥ 99.8%) was from VWR. Diethyl ether (stabilized, ≥ 97.5%), triethylamine (≥ 99.5%), trifluoroacetic acid (TFA, 99%) and N-(2-aminoethyl)maleimide trifluoroacetate (AEMI, 99.5%) were from Sigma. Coupling reagents 1,3-dicyclohexylcarbodiimide (DCC) and N-hydroxysuccinimide (NHS) were synthesis grade from Sigma. All solvents were used as received.

For GPC eluent preparation, the following materials were used: THF (HPLC-grade) were obtained from Honeywell and Fisher Scientific, DMF (HPLC-grade) from Sigma, LiBr (analytical grade) from Merck, TBAB (99.0%) from Sigma-Aldrich and NaNO_3_ (GPR Rectapur) from VWR. Narrow MWD standards were purchased from Polymer Standard Service.

### Characterization of polymer samples

The commercial PCL-PEG samples were characterized by GPC, and identities were confirmed by ^1^HNMR (data not shown). Number-average molecular weights (M_n_) of 12.1, 16.3, and 14.9 kDa and weight-average molecular weights (M_w_) of 14.6, 19.5 and 34.3 kDa were obtained by GPC for PCL-PEG-Me, PCL-PEG-COOH and PCL-PEG-MAL, respectively. The COOH- and methyl-endcapped polymers were of low dispersity at Ð= 1.2 whereas the maleimide-endcapped polymer had a wider MWD (Ð = 2.31).

### Synthesis of PCL-PEG-NHS

PCL-PEG-COOH (100 mg, 1.0 equivalents) and NHS (9.87 mg, 12.5 eq.) were placed in a dry RBF containing a stirring magnet. The flask was sealed and flushed with argon. DCC (14.15 mg, 10.0 eq.) was weighed to a dry glass vial and dissolved in 0.20 mL DCM. To the flask, 0.48 mL DCM and 0.25 mL DMF were added by syringe and stirring was started. When everything had dissolved, DCC solution was added under Ar flow. The mixture was stirred for 22 h at RT. Precipitated solids were removed by syringe filtration through a 0.45 μm PTFE filter (VWR). The solution was precipitated in 13.5 mL of ice cold Et_2_O / MeOH (2:1) mixture in a centrifuge tube. The tube was transferred to -20 °C for 20 min and a white precipitate formed. The mixture was centrifuged for 5 min at 4000 g, + 4 °C and the supernatant was decanted away. The pellet was washed twice with ice cold Et_2_O / MeOH mixture and flushed with dry N_2_ for 3 h to evaporate solvents. A white solid was obtained with 84% gravimetric yield and used without further characterization.

### Synthesis of PCL-PEG-maleimide

Into a glass vial containing the obtained PCL-PEG-NHS (85.18 mg, 1.0 eq.), AEMI (8.98 mg, 6.0 eq.), anhydr. DCM (0.30 mL) and anhydr. DMF (0.20 mL) were added, the solids were dissolved by agitation, and the solution was transferred to a dry RBF flushed with Ar. Under magnetic stirring, 8.0 µL of Et_3_N was added and pH was checked by pipetting 5 µL of the reaction mixture onto a pH indicator paper wetted with H_2_O, giving a pH value of 9–10. After stirring for 3 h at RT under Ar, the reaction was stopped by adding 6 µL of TFA. The product was precipitated by adding the solution dropwise to 13.5 mL of ice cold Et_2_O / MeOH (2:1) mixture in a centrifuge tube. A white precipitate formed, and it was centrifuged and washed twice with ice cold Et_2_O / MeOH as above. The pellet was flushed with dry N_2_ for 3 h to evaporate solvents. A white solid was obtained with a yield or 75.4 mg (88.5%).

Maleimide content was analyzed spectrophotometrically (UV-1600 PC, VWR) by absorbance at 300 nm in anhydrous DMF and calculated using a standard calibration of AEMI. The absorbance data indicated 62.4% conversion from PCL-PEG-COOH to PCL-PEG-maleimide (42.9 µmol maleimide in 1.0 mg product). The presence of maleimide in the sample was also verified by ^1^H NMR (500 MHz, CDCl_3_), showing a split signal at 6.7 ppm.

### Synthesis of peptide-polymer conjugates (pre-conjugation approach)

To conjugate the peptide and control fluorophore to polymer, 20 mg (1.3 µmol, 1 eq.) of PCL-PEG-maleimide and 1.1 µmol (0.8 eq, 0.51 mg of 5-FAM-Cys or 2.06 mg of 5-FAM-Cys-TT1) were weighed in glass vials, dissolved in 0.40 mL of anhydrous DMF and allowed to react in closed vials at RT overnight, protected from light. The products were precipitated as nanoparticles by adding 1.6 mL of H_2_O, purified by dialysis against 500 mL H_2_O (50 kDa MWCO, Spectra/Por 7) for 8 h with one water change at 3.5 h, and lyophilized. PCL-PEG-FAM was obtained as a slightly green solid and PCL-PEG-FAM-TT1 as a strongly orange-colored solid with yields of 15.2 mg (74.7%) and 19.53 mg (89.1%), respectively. To evaluate success of conjugation, analysis by gel permeation chromatography (GPC) was performed with dual detection (UV absorbance + refractive index).

### Preparation of nanoparticles by microfluidic nanoprecipitation

Microfluidic nanoprecipitation [29, 30] was performed using a glass capillary flow-focusing type microfluidic mixer as described in our previous work [25]. First, polymers were dissolved in acetone at 10 mg/mL and injected into the inner channel of the device at 30 mL/h, and deionized water was injected to the outer channels at 120 mL/min using syringe pumps. Filters (0.45 μm, Nylon, PALL) were included in the syringes to remove any particulate matter. During injection, the device was immersed in a + 65 °C water bath. The injection results in rapid mixing of the two solutions and formation of nanoparticles inside the device. The dispersions were collected from the device into suitable media and purified by CUF using 100k MWCO tubes (Amicon Ultra-15, Merck) to remove organic solvent.

### Preparation of nanoparticles by batch nanoprecipitation

Mixtures of polymers were dissolved in anhydr. DMF at 5–10 mg/mL and added dropwise into the aqueous phase (deionized water with 0.25% P188) in a glass vial at R.T. under moderate magnetic stirring, to a final aqueous-organic ratio of 4:1. In the colloidal stability experiment in Supplementary information, alternative aqueous solutions were used, as detailed therein. For pre-conjugated samples and control samples, the buffer was exchanged to PBS 0.25% P188 pH 7.4 by CUF (100k) over three washing cycles, and NPs were collected at 5 mg/mL final concentration. For post-conjugated samples, the reaction was performed as detailed below.

### Conjugation of peptide to nanoparticles (post-conjugation approach)

The NPs were prepared using mixtures of reactive (maleimide terminated, PCL-PEG-MAL) and non-reactive block copolymers (methyl ether or acid terminated, PCL-PEG-Me or PCL-PEG-COOH) to control the ligand density and the ζ-potential of NP’s surfaces. The dilution of reactive polymer with similar non-reactive material is a common method to control the number of ligands per nanoparticle [10, 26, 31]. The acid-terminated PCL-PEG-COOH was included, because in our earlier optimization work, it increased the encapsulation of a hydrophobic, basic drug and resulted in small, homogeneous and spherical NPs [25].

In the initial experiments (Figs. 1 and 2), peptide conjugation was performed as follows: The peptide FAM-Cys-Ahx-AKRGARSTA-NH_2_ (where FAM = 5-carboxyfluorescein, Cys = cysteine, Ahx = aminohexanoic acid spacer) was first dissolved in the conjugation buffer (HEPES pH 7.0 or PBS pH 7.4). To reduce any disulfides to free thiols, the peptide solution was treated with 4-fold molar excess of TCEP prior to conjugation. After formation of NPs by the microfluidic nanoprecipitation method, the dispersant was immediately changed to the conjugation buffer by CUF, the dispersion was immediately transferred to a new vial and TCEP-activated peptide (at thiol-maleimide ratio 1:2, 1:1 or 3:1) was added dropwise under stirring. The reaction was continued for 1 h in the dark at R.T. and free peptide was removed by CUF (100k). During the free peptide removal, adsorption of the peptide or peptide-conjugated NPs onto the filtration membrane was observed, based on color change of the filter membrane from white to orangeA. Aliquots were taken at different stages of the process for dynamic light scattering (DLS) measurement.

In the final experiments (Figs. 5–9), in-house synthesized PCL-PEG-MAL was diluted with PCL-PEG-Me and PCL-PEG-COOH, and the NPs were prepared by batch nanoprecipitation method. Peptide post-conjugation was performed immediately after the nanoprecipitation step, without removal of the residual organic solvent. A hydroxyl-terminated version of the peptide (FAM-Cys-Ahx-AKRGARSTA-OH) or the control ligand (FAM-Cys) was dissolved in PBS 0.25% P188 (pH 7.4) and immediately added dropwise to the NP dispersion under stirring and allowed to react at RT for 1 h in the dark. The free peptide was removed by CUF (100k) over three dilution-concentration cycles in PBS 0.25% P188 (pH 7.4) at 2500 g, + 20 °C and NPs were collected at 5 mg/mL final concentration.

### Physicochemical characterization of the NPs

Particle size and zeta (ζ)-potential of NPs were measured by DLS on a Zetasizer Nano ZS (Malvern) in disposable folded capillary cells (DTS1070, Malvern) in 0.1× PBS 0.025% P188 (pH 7.4; 0.67 mM phosphate, 15 mM NaCl). The measurements were in triplicates conducted at 173° scattering angle at + 25 °C.

For electron microscopy studies, NPs in PBS 0.25% P188 were first dialyzed (50 kDa MWCO, Spectra/Por 7) overnight at RT against deionized water to remove salts and then filtered through 0.45 μm cellulose acetate filters (VWR). Formvar/carbon coated copper grides (200 mesh, Electron Microscopy Sciences) were rendered hydrophilic by glow discharge treatment for 45 s at 25 mA (K100X, Emitech). TEM specimens were prepared by applying 5 µL of sample on the grid for 45–60 s, blotting the drop away filter paper, and immediately applying a drop of 1% uranyl acetate. After 45–60 s, the excess stain was carefully blotted away with filter paper and the grids were dried in ambient air, followed by imaging at 100 kV acceleration voltage on a HT7800 TEM (Hitachi) instrument.

The amounts of Cys-FAM and Cys-FAM-Ahx-AKRGARSTA-OH in NPs were quantified spectrophotometrically; NPs were dispersed in 1× PBS 0.25% P188 (pH 7.4) at 1.0 mg/mL, and absorbance at 488 nm was measured on a white walled clear bottom polystyrene 96-well plate (*n* = 3, 150 µL per well) with Varioskan LUX plate reader (Thermo Fisher). Due to different particle sizes, manual scattering correction was performed to the absorbance spectra to compensate for background scattering by NPs. The ligand amounts were calculated using a calibration curve of Cys-FAM in the same buffer. Average values and standard deviations were obtained by combining absorbance data from all prepared NP samples. Fluorescence intensities of NPs dispersions were measured on the same plate, using excitation at 488 nm and emission at 512 nm (*n* = 3, 150 µL per well, top optics).

### Polymer solubility determination by turbidity measurement

To evaluate the suitability of THF/water mixtures as GPC eluents for these polymer-peptide conjugates, the solubility of PCL-PEG at different water/THF ratios was tested spectrophotometrically by measuring increase in solution turbidity due to micellization upon water addition. To begin, 750 µL of 10 mg/mL PCL-PEG-Me 10k-2k in anhydrous THF was placed in a quartz cuvette (HELLMA) with a magnetic stir bar and absorbance values at 400 and 600 nm were recorded on a spectrophotometer (UV 1600-PC, VWR). Deionized water was then added in 75 µL aliquots, followed by pipette mixing and magnetic stirring for 2 min between each addition. The absorbance values were recorded after each addition step, and this was continued until a total of 300 µL of water was added and a sharp increase in sample turbidity was observed. Then, re-solubilization of the polymer was achieved by adding 75 µL aliquots of THF with the same protocol until no appreciable absorbance compared to blank was seen. The data was evaluated by plotting absorbance as a function of water content.

### GPC analysis

For GPC analysis, all polymer samples were dissolved in eluent at 1–2 mg/mL and filtered through 0.22 μm PTFE syringe filters prior to injection. Molecular weights of base polymers were analyzed in THF on a system consisting of a Waters 515 HPLC pump, Biotech DEGASi GPC Degasser, Waters 717 plus Autosampler, Waters 2487 Dual λ Absorbance Detector and Waters 2410 Differential Refractometer, with Waters Styragel HR2, HR4, and HR5E (7.8 × 300 mm) columns and a guard column. Eluent flow rate 0.800 mL/min and column temperature 30 °C were used. Molecular weight calibration was performed with polystyrene standards.

Analyses in DMF 50 mM LiBr were performed on an Agilent 1260 Infinity II GPC-SEC-MDS system consisting of 1260 Infinity II Isocratic Pump, 1260 Infinity II Degasser, 1260 Infinity II Vialsampler, 1260 Infinity II Multicolumn Thermostat MCT, 1260 Infinity II Variable Wavelength Detector with standard flow cell, 1260 Infinity II Fluorescence Detector, and Agilent triple detection suite with 1260 GPC/SEC MDS Refractive Index Detector, 1260 GPC/SEC MDS Viscometer Detector and 1260 GPC/SEC GPC MDS Dual Angle Light Scattering Detector. For separation Waters Styragel HT5, HR4 and HR2 (7.8 × 300 mm) columns thermostated at 40 °C were used. Flow rate was 0.800 mL/min. Molecular weight calibration was performed with PMMA standards.

Analyses in THF/water/TBAB were performed on a Waters Acquity APC system on a Novema Max 1000 Å column (PSS). The system consisted of Acquity Column Manager – S, Sample Manager – pFTN, p – Isocratic Solvent Manager, Acquity RI Detector and Acquity TUV Detector. Flow rate was set to 0.800 ml/min and column was thermostated at 30 °C. The eluent used was 90:10 THF: water containing 1 g/L TBAB. Water was purified using Elga Purelab Ultra.

### Cell-free p32 protein binding assay

The binding assay was performed using magnetic Ni-NTA agarose beads, with three replicate assay tubes per sample. The beads (10 µL of 5% v/v suspension) were washed twice with Binding and Washing Buffer (BWB, 1× PBS with 5 mM imidazole pH 7.4) and incubated with 15 µg of 6xHis-tagged p32 for 1 h under gentle rocking at RT. The beads were washed with 1% BSA and redispersed in 0.1% BSA in BWB.

Prior to mixing with the beads, NP samples were first diluted in BWB to 5 µM ligand concentration by fluorescence (ca. 1 mg/mL polymer). The beads (100 µL) were incubated with NP samples (100 µL) for 1 h at RT in the dark, and then washed 5× with 200 µL BWB. Finally, 100 µL of elution buffer (400 mM imidazole in 1× PBS pH 7.4) was added to the beads to break the NTA-6xHis bonds, the eluates were collected and fluorescence measured at 488/525 nm (Varioskan LUX, Thermo) on a white-walled clear bottom polystyrene 96-well plate (Corning).

### Data analysis

All plotting and statistical analyses were performed using Origin Pro 2024 R1 software v. 10. Statistical significance of the protein binding assay results was determined by pairwise comparison using Tukey’s significance test, with *p* ≤ 0.05 defined as the significance limit.

## Results and discussion

### Post-conjugation of peptide resulted in agglomeration during post-processing

Conjugation of the labelled peptide to maleimide-functionalized NPs in HEPES buffer, with subsequent purification by CUF, significantly increased the polydispersity index (PDI) and average hydrodynamic diameter (d_H_) of the NPs (Fig. 1A-B), and these changes corresponded with visually detectable agglomeration in the respective samples. The increase was smallest in NPs prepared from a blend of PCL-PEG-COOH and PCL-PEG-MAL (80:20) and highest in samples prepared PCL-PEG-Me and PCL-PEG-MAL (80:20). The higher colloidal stability of PCL-PEG-COOH containing NPs may be related to negative charge leading to electrostatic stabilization.

Similar conjugation was attempted in isotonic phosphate buffer (PBS, pH 7.4, 150 mM NaCl) for mixtures of PCL-PEG-Me and PCL-PEG-MAL (1:2, 1:1 and 3:1) with additional particle size measurements taken before conjugation. The data (Fig. 2A-B) as well as visual examination of the samples indicated that particle size and PDI increased already during the first CUF step and continued further during the peptide conjugation step. The colloidal stability of NPs was inferior in isotonic PBS compared to the salt-free HEPES, as expected due to higher ionic strength of the PBS. The results showed that the use of a dispersion stabilizer is needed to allow the purification and dispersion of these NPs in isotonic buffers, which is necessary for their intended use as intravenously administered drug delivery systems.

### Stabilization with poloxamers reduces agglomeration during post-processing

Closer investigation of the colloidal stability of methyl-endcapped PCL-PEG 10k-2k NPs was performed and is detailed in the Supplementary information. The data showed that the NPs suffered from poor stability in PBS when strongly agitated by hand or subjected to CUF at 2500–4000 g. To stabilize the NPs, poloxamer 188 non-ionic surfactant (P188) was included in the buffer and it was found to significantly reduce agglomeration of PCL-PEG-Me NPs under these conditions (Figures S1 and S2). The use of this stabilizer was implemented at 0.25% (w/v) for all subsequent NP-preparations and purifications. Data from the stabilization experiment also suggested that the agglomeration tendency was decreased when a polymer with a shorter PCL block was used, and agglomeration tendency was highest when the PCL-PEG-MAL polymer with high M_w_ and dispersity was included in the mixture. This can be due to reduced PEG tethering density on NP surface, or simply due to increased sedimentation rate during CUF caused by larger particle size.

### Characterization of the synthesized conjugates

To obtain a maleimide-functionalized polymer with a narrow MWD, we synthesized PCL-PEG-MAL in-house using the previously analyzed PCL-PEG-COOH precursor, as detailed in Materials and Methods, and reacted it further with either a control fluorophore (FAM-Cys) or a hydroxyl-terminated labelled peptide FAM-Cys-Ahx-AKRGARSTA-OH, followed by purification and lyophilization.

**Fig. 3:**
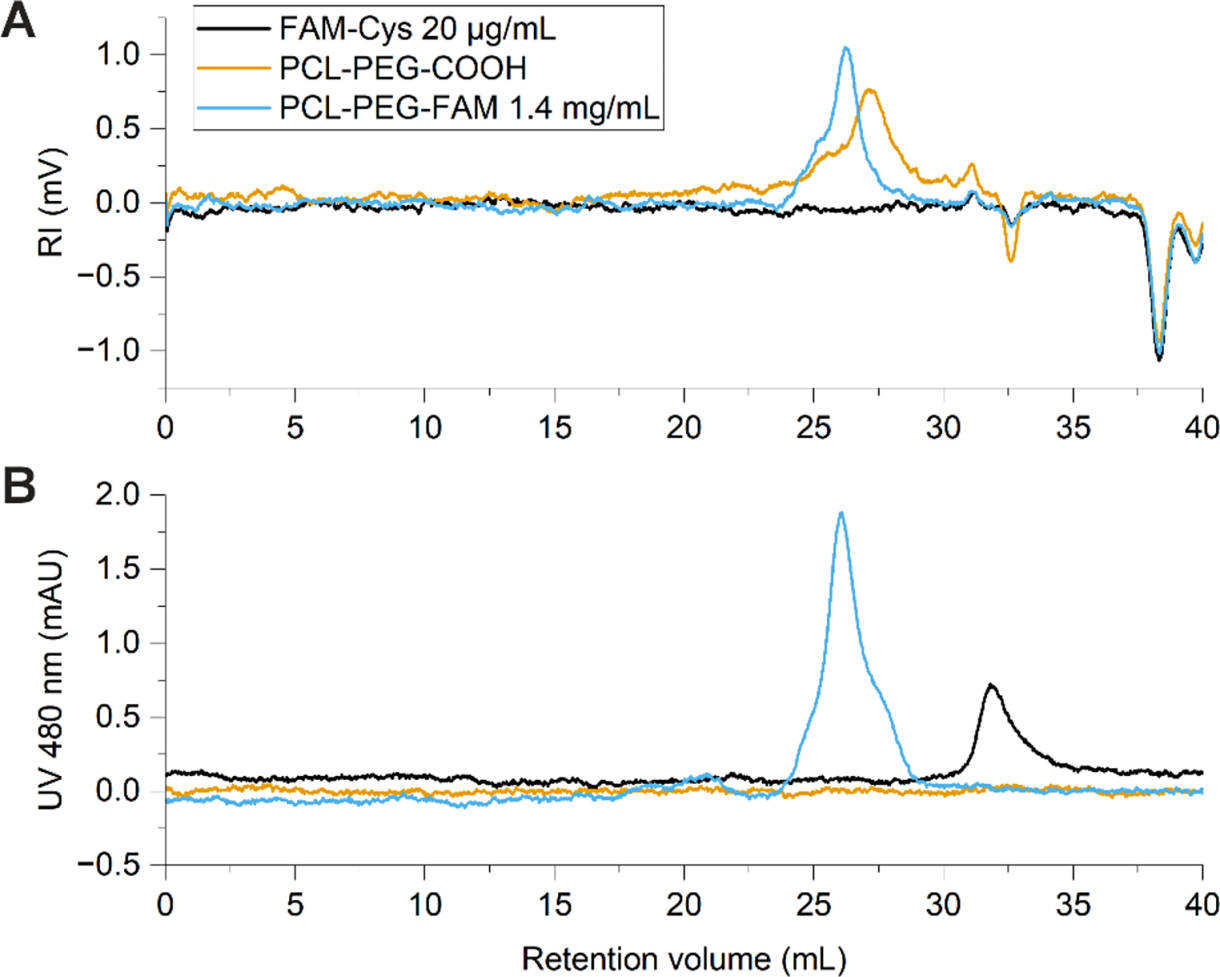
GPC of the PCL-PEG-FAM conjugate, and the relevant controls, non-functionalized polymer (PCL-PEG-COOH) and free ligand (FAM-Cys). The samples were run on a Styragel column set with 50 mM LiBr in DMF as the mobile phase and detection by RI (**A**) and absorbance at 480 nm (**B**)

The fluorophore-polymer conjugate PCL-PEG-FAM and its building blocks were characterized by GPC in DMF + 50 mM lithium bromide (LiBr) on a set of Styragel HT5, HR4 and HR2 columns (Waters) and molecular weights were determined against poly(methyl methacrylate) (PMMA) standards. The refractive index (RI) channel peaks at 26–28 mL for PCL-PEG-COOH and PCL-PEG-FAM correspond to the polymer signal (Fig. 3). A strong absorbance signal at 480 nm is visible for PCL-PEG-FAM at this retention volume which indicates successful conjugation. The free peptide FAM-Cys gives an absorbance peak at 32 mL, and this peak cannot be found in the conjugate sample, which indicates the lack of free FAM-Cys in the purified conjugate. The data suggests the reaction and purification cycles increased the average molecular weight from M_n_ = 13.3 kDa and M_w_ = 17.8 kDa (Ð = 1.34) for the starting material PCL-PEG-COOH to M_n_ = 20.2 kDa and M_w_ = 24.7 kDa g/mol (Ð = 1.22) for the conjugate. The increase can be attributed to loss of low-molecular weight species by the multiple precipitation purification and dialysis steps.

Analyses of the free peptide and the polymer-peptide conjugate PCL-PEG-TT1 were attempted using the same method. However, even though the materials were well soluble in the eluent, no peaks were detected for any of the peptide-containing samples. A slowly decreasing, persistent background signal was observed in UV-channel during subsequent column washing cycles, which suggests binding of peptide and peptide-conjugates to the column material, possibly caused by the cationic nature of the peptide.

### Attempts at GPC analysis of the polymer-peptide conjugate

A more suitable GPC method for analyzing the peptide-polymer conjugate was needed to confirm covalent conjugation. To avoid the cationic side chains of the peptide from binding to the stationary phase, we tested an amine-functionalized acrylate copolymer column (NOVEMA^®^ Max, PSS), which is intended for the analysis of cationic polymers. Using this column, good separation of the peptide from a solution of P188 was obtained in aqueous mobile phase (0.10 M NaNO_3_ + 3% acetonitrile, Figures S4 and S5). To see if the terminal acid group of PCL-PEG-COOH could result in undesired ionic interactions between the base polymer and the cationic column, a control experiment was performed in aqueous mobile phase (0.10 M NaNO_3_ + 3% acetonitrile) for acid- and hydroxyl-terminated PEGs. Well resolved peaks were obtained and no significant difference in elution volumes were between PEG-COOH and PEG-OH of similar molecular weight (Figure S3).

However, to allow separation of free peptide and PCL-PEG-TT1 conjugate, it is necessary to find a solvent mixture that can dissolve all sample components and does not degrade or dissolve the stationary phase. Different chemical natures of the peptide, PEG and PCL blocks make it challenging to find a suitable solvent system and stationary phase combination, and stability concerns limit the use of high temperatures or extreme pH for solubility improvement. Compatibility of the column with DMF was not known and the only organic solvents, for which compatibility information was available, were acetonitrile, methanol, and tetrahydrofuran (THF). Of these solvents, only THF can dissolve PCL at RT. Since pure THF does not dissolve the peptide, the solubilities of peptide and base polymer were first tested in mixtures of THF and water.

**Fig. 4:**
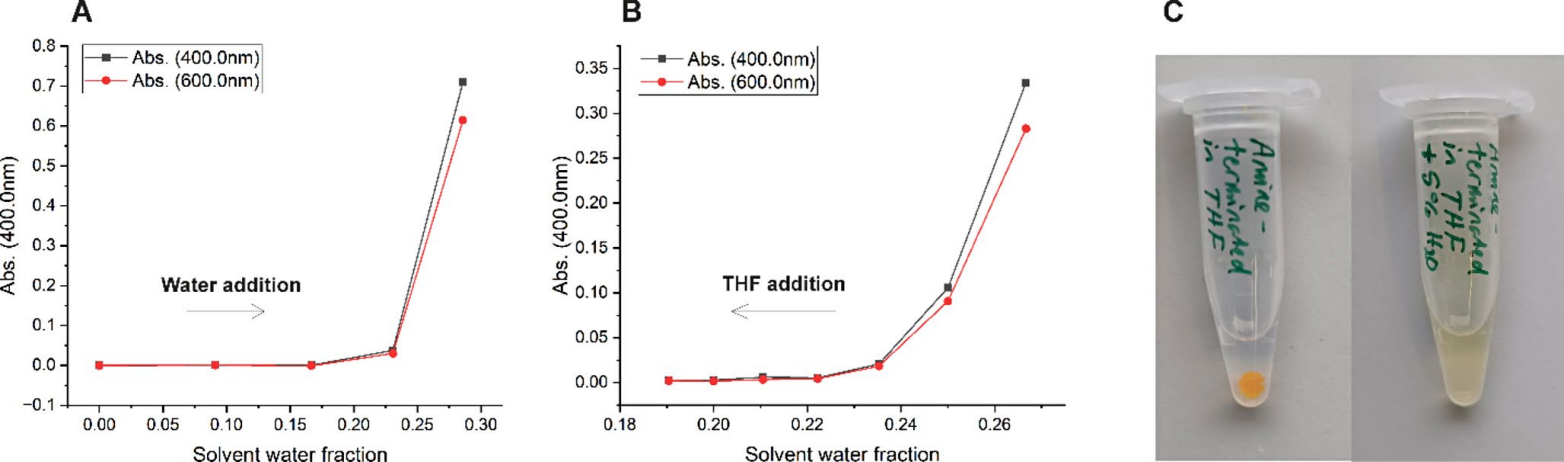
Solubility tests for sample components to find a suitable GPC mobile phase. Turbidity plots of PCL-PEG-Me 10k-2k solution in THF-water mixtures were prepared to evaluate critical water fraction below which the polymer remains molecularly dissolved (**A-B**), and visual solubility experiments were performed to minimum water content for solubilizing the peptide (**C**)

Turbidimetric measurements were performed for polymer solutions at different water/THF ratios, and the data indicated that PEG-PCL remains fully dissolved below 20% water content (Figue 4 A-B). Visual solubility tests for an amine-terminated version of the peptide showed that as low as 5% of water in THF is sufficient to dissolve the peptide in relevant concentrations (Fig. 4C). Accordingly, 10% (v/v) water content in THF was chosen for the mobile phase. To disperse ionic interactions, tetrabutylammonium bromide (TBAB) was added to the mobile phase at 1 g/L. Samples were dissolved in the eluent and filtered using PTFE syringe filters before analysis. UV/VIS-absorbance spectra of relevant samples were measured in the eluent to determine proper wavelengths for detection.

**Fig. 5:**
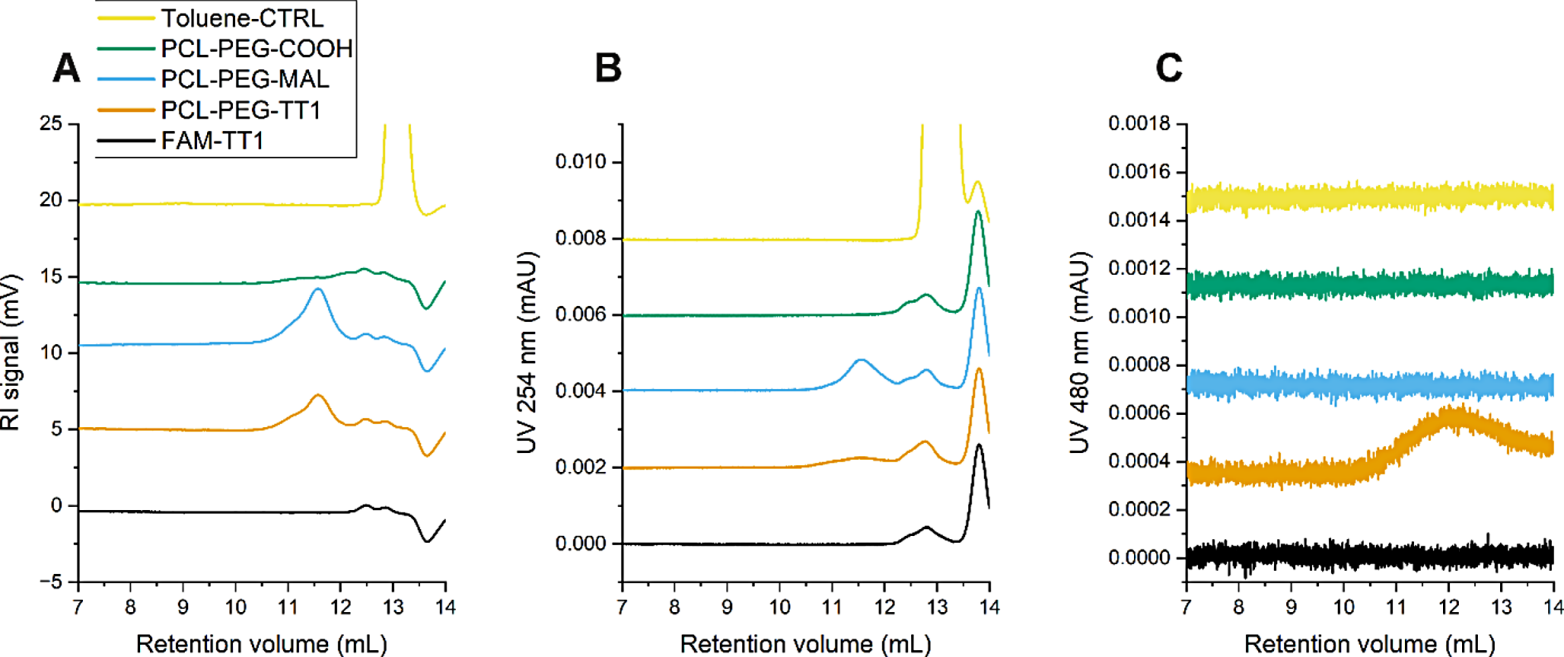
GPC of PCL-PEG-TT1 conjugate and the relevant controls with detection by RI (**A**), absorbance at 254 nm (**B**) and absorbance at 480 nm (**C**). Samples were run on a Novema Max column in THF/water with 0.1% of TBAB

Figure 5 shows the GPC data obtained using the THF/water/TBAB mobile phase on the cationic column. The RI signals of PCL-PEG-MAL and PCL-PEG-TT1 show an expected elution profile at 11–12 min and no additional peaks compared to the small molecule control (toluene in eluent). PCL-PEG-MAL shows a strong absorption in UV-channel 254 nm at polymer retention time, confirming the presence of a maleimide group. The overlapping peaks in UV and RI channels at 11–12 mL for PCL-PEG-TT1 indicate that covalent conjugation of the labelled peptide FAM-Cys-Ahx-AKRGARSTA-OH was successful (Fig. 5A-C). However, the FAM-specific UV-signal spreads to retention volumes far above 12 mL, showing that the conjugate does not elute in a single sharp peak (Fig. 5C) and, therefore, estimation of molecular weight is not possible.

The base polymer PCL-PEG-COOH gave a weak RI signal at 11–13 mL (Fig. 5A), which seems to be spread to higher retention volumes. This may be due to reduced hydrodynamic radius of the polymer coil in the presence of water and salt, or due to ionic interactions with the cationic column material and the terminal acid group, even though no such interactions were seen in the aqueous control experiment described above. The most likely cause is diminished solubility of PEG to the eluent due to addition of salt and THF being a poor solvent for pure PEG. Poor solubility of the relatively short PEG-block is likely to cause adsorption on syringe filters and column packing material, but modification of the functional end increases the solubility to such an extent that GPC analysis of the modified polymers is possible. Our efforts for additional relative calibration using PEG standards failed because they were insoluble in the eluent used. Limited solubility of PEG in the eluent was confirmed in additional control experiments.

Reduction of the TBAB or water concentrations, or the inclusion of a small amount of organic acid in mobile phase to protonate the COOH-terminus can be considered to improve the solubility and elution behavior of this polymer. With this solvent system, no signal was detected in any channel for the free peptide, even though the peptide was easily dissolved in the eluent. This can potentially be caused by adsorption of the peptide onto the syringe filters during sample preparation or onto components of the chromatography system.

Qualitatively, the data suggest successful maleimide and peptide functionalization reactions of the polymer, based on the co-appearance of UV-signal at the polymer retention volume. However, since well resolved peaks were not obtained for PCL-PEG-COOH or the free peptide, further development of the method would be necessary to enable reliable quantitative analysis of the ratio of conjugated to free peptide and the final molecular weight of the conjugate.

GPC is a powerful method for analyzing biomolecular conjugates in their molecularly dissolved state and several reports are available for the GPC analysis of PEG-biomolecule conjugates, which are water soluble [17, 19, 32, 33]. For instance, Decked and Maynard analyzed the MWD of PEG-protein conjugates in 0.1 M LiBr in DMF on a set of styrene-divinylbenzene copolymer columns [33]. However, a limited amount of literature can be found on the analysis of water-insoluble polymer-peptide conjugates. Trimaille et al. attempted to analyze polystyrene-peptide conjugates in pure THF, and reported that the 9-amino acid peptide GGGWIKVAV was insoluble in THF, but at high enough molecular weights of polystyrene, the conjugate became soluble and the analysis could be performed [18].

To enable size-based separation of free peptide from PCL-PEG-peptide conjugates, the binding of peptide to different stationary phases in different solvent mixtures and pH, as well as the compatibility of the stationary phases with polar aprotic organic solvents like DMF, N-methyl(2-pyrrolidone) or dimethylacetamide should be evaluated. Furthermore, the hydrodynamic radii of the sample components can be drastically affected by the mixed organic/aqueous mobile phase, and the effect of this on the retention volume should be evaluated to enable MWD analysis against suitable standards. If UV detection is to be used, including a chromophore in the peptide ligand is necessary to allow detection and quantification. Here, the used peptide was labelled with FAM. However, any UV-active groups like phenylalanine, tyrosine or tryptophan amino acids can be included in the peptide moiety to allow detection as long as they do not affect the pharmacology or induce toxicity.

### Particles conjugated with labelled peptide show low fluorescence yield per ligand amount

NPs were prepared by a batch nanoprecipitation method [34] either from the pre-synthesized conjugates or by including PCL-PEG-MAL in the polymer mixture polymer and reacting with peptide after nanoprecipitation as detailed in Materials and Methods. In these experiments, no discoloration of the CUF membrane was observed during the purification step, and this was attributed to the use of P188 in the buffer, which seemed to effectively prevent peptide adsorption. When PCL-PEG-COOH was used as the diluent polymer, no visible particle sedimentation or agglomeration occurred during the reaction and washing steps. However, when PCL-PEG-Me was used as diluent in post-conjugated samples, a small amount of material sedimented onto the filter during the CUF process and could not be redispersed as NPs.

**Fig. 6:**
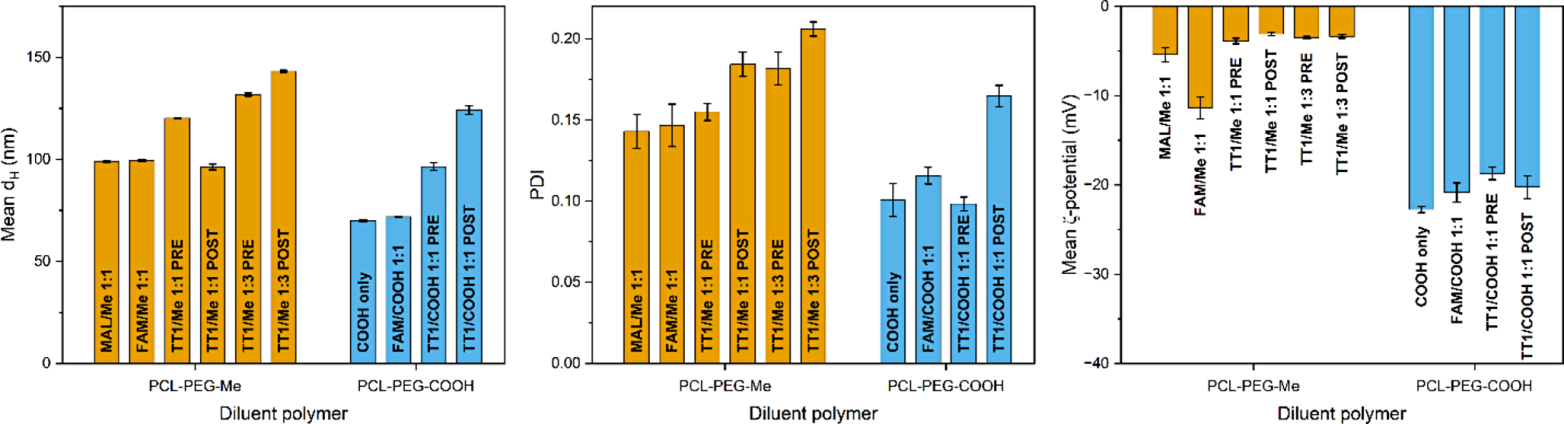
DLS/ELS characterization results of the NPs prepared from mixtures of functional and inactive polymers with peptide attachment before or after nanoprecipitation. Mean hydrodynamic diameter (d_H_), polydispersity index (PDI) and ζ-potential values were recorded in diluted PBS (pH 7.4, 0.67 mM phosphate, 15 mM NaCl). Averages values ± SD from three repeat measurements are shown

In Fig. 6, DLS data show the formation of homogeneous dispersions (PDI ≤ 0.2) with average hydrodynamic diameter (d_H_) values ranging from 49.0 ± 0.2 to 143.2 ± 0.5 nm, depending on the polymer mixture used. Cys-FAM conjugation to the polymer did not affect particle size or PDI to a practically significant degree. However, peptide conjugation increased the particle size in all samples except the TT1/Me 1:1 ratio post-conjugated sample. Post-conjugated particles showed a larger particle size and PDI than pre-conjugated samples with the exception of the TT1/Me 1:1 ratio samples. For example, the PCL-PEG-COOH diluted samples showed an increase in d_H_ from 70 nm (PDI 0.10) in non-conjugated to 72 nm (PDI 0.12) in FAM-conjugated, 96 nm (PDI 0.10) in peptide pre-conjugated and 124 nm (PDI 0.17) in peptide post-conjugated sample.

Conjugation was also associated with small changes in particle ζ-potential when measured in diluted PBS (pH 7.4; 0.67 mM phosphate, 15 mM NaCl). For example, NPs with 1:1 mixture of PCL-PEG-Me and PCL-PEG-MAL gave an average ζ-potential of -5.4 ± 0.8 mV, whereas mixtures of PCL-PEG-Me and PCL-PEG-FAM gave an average value of -11.6 ± 1.2 mV, as expected based on the multiple carboxylic acid groups in 5-FAM. Attachment of the peptide caused minor increases in ζ-potential. For example, a 1:1 mixture of PCL-PEG-COOH and pre-conjugated PCL-PEG-TT1 showed an average ζ-potential of -18.7 ± 0.7 mV compared to -22.7 ± 0.4 mV obtained for PCL-PEG-COOH NPs. Only minimal differences in ζ-potential were seen between the pre- and post-conjugated samples.

Fluorescence and absorbance spectra of NPs were measured in the dispersing buffer at 1.0 mg/mL and the amounts of ligand attached per mass unit of NPs and the conversion degrees of base polymer to conjugate were calculated based on FAM-specific absorbance at 488 nm. For PCL-PEG-FAM, a final ligand content of 23.5 ± 3.0 nmol/mg was obtained, which corresponds to 41–54% conversion of base polymer to PCL-PEG-FAM with M_n_ = 20.2 kDa. For PCL-PEG-TT1 conjugate, a ligand content of 27.3 ± 2.7 nmol/mg was obtained, corresponding to 50–61% conversion assuming the final molecular weight is similar to that of PCL-PEG-FAM.

**Fig. 7:**
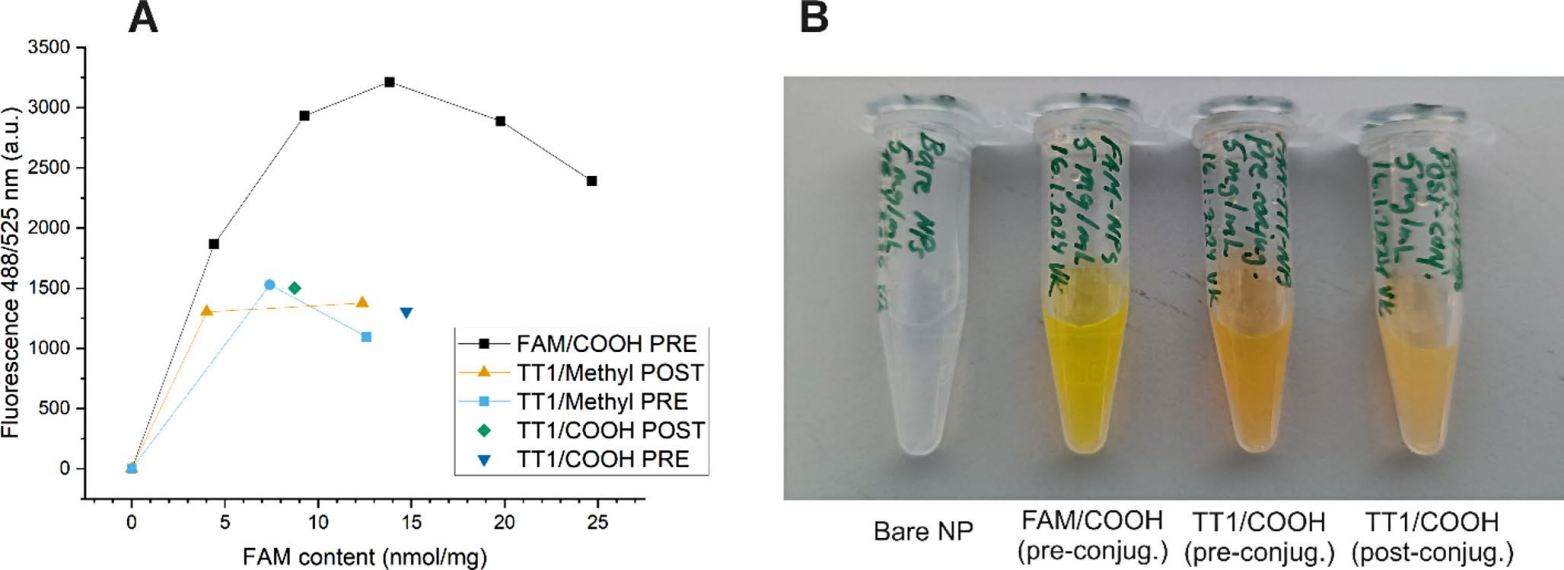
NPs were prepared at different ratios of functional polymers and methyl- or COOH-terminated inactive polymers. (**A**) Fluorescence intensity of the NPs prepared from different polymer mixtures as a function of ligand amount in nanoparticles. All samples were dispersed in 1× PBS 0.25% P188 (pH 7.4) at 1.0 mg/mL NP concentration. Fluorescence was measured at 488/525 nm and ligand amount in NPs was determined based on absorbance at 488 nm. (**B**) Photographs of NP dispersion prepared from mixtures of functional and using either pre-conjugated material (pre) or performing the peptide conjugation after nanoprecipitation (post)

Figure 7A shows the fluorescence of the NPs as a function of the amount of ligand attached at 1.0 mg/mL NP concentration in the pH 7.4 buffer. It is well-known that FAM exhibits reduced net fluorescence at high concentrations in aqueous buffers due to self-quenching effect, and this becomes visible also in Fig. 7A as the amount of ligands per NP is increased. Interestingly, For PCL-PEG-FAM NPs, a maximum fluorescence intensity of approximately 3200 units is reached at 14 nmol ligand per 1 mg NPs, whereas the FAM-LinTT1-conjugated NPs reach a lower maximum of 1000–1500 units at ca. 5 nmol/mg with no further increase in fluorescence upon increasing the ligand amount.

When dispersed in pH 7.4 buffer, the non-conjugated NPs appeared colorless, the FAM-Cys conjugated samples fluorescent yellow-green and peptide conjugated samples orange in color (Fig. 7B). Upon storage of aqueous solutions of the free labelled peptide, we noticed a similar change of color from green to orange and a reduction in fluorescence. The green color (associated with fluorescence) of the peptide solution was restored upon treatment with tris(2-carboxyethyl)phosphine (TCEP), which means that the loss of fluorescence in the case of free peptide was due to dimerization through the cysteine thiols, which brings two fluorophores close together, causing self-quenching.

This different visual appearance of NPs in conjunction with the fluorescence data suggests that a subpopulation of the ligands in FAM-LinTT1-conjugated particles are not able to fluoresce or exhibit self-quenching. Theoretically, this can be caused by conjugated ligands facing toward the interior of NPs as opposed to being exposed toward the aqueous medium, or by the presence of encapsulated non-conjugated peptide, as encapsulated labelled peptides will be in close vicinity to each other. Self-quenching of fluorescein when encapsulated in high concentration inside PCL-PEG vesicles was also reported elsewhere [35].

The number of ligands per NP can be estimated based on the measured FAM absorbance per mass unit of NPs and the mass of single NP. A rough estimate for the average particle mass $\:{M}_{NP}$ can be obtained based on average diameter $\:d$ and polymer density $\:\rho\:$ (Eq. 1), and the aggregation number can be estimated based on the average particle mass and $\:{M}_{n}$ (Eq. 2) [36].

1 $$\:{M}_{NP}=\frac{1}{6}\rho\:\pi\:{d}^{3}$$

2 $$\:{N}_{agg}=\frac{{{N}_{A}M}_{NP}}{{M}_{n}}=\frac{{N}_{A}\rho\:\pi\:{d}^{3}}{6{M}_{n}}$$

Using the density of amorphous solid polycaprolactone [37] of $\:\rho\:$ =1.081 g/cm^3^, *d* = 0.10 μm and *M*_*n*_ = 2.02 × 10^4^ g/mol, an aggregation number of 1.7 × 10^4^ polymer chains per particle is obtained. At a ligand amount of 5.0 nmol/mg, this corresponds to ca. 1700 ligands per NP, which is expected to be sufficient to achieve strong multivalent binding. For example, Montet et al. studied multivalent effects with iron oxide NPs (diameter ca. 30 nm) conjugated with peptides containing a cyclic RGD motif, and discovered significant NP uptake to αVβ3-integrin expressing endothelial and tumor cells at as low as 20 peptide ligands per NPs [38]. In a previous work with the LinTT1 peptide, reported by Simón-Gracia et al., PEG-PCL polymersomes of d_H_ ≈ 100 nm with ca. 3.7 × 10^4^ peptide ligands per NP showed significant binding to p32 protein, were taken up by breast cancer cell lines overexpressing the protein, and showed tumor accumulation and retention in vivo [26].

### Both pre- and post-conjugated particles bind their protein target in vitro

**Fig. 8:**
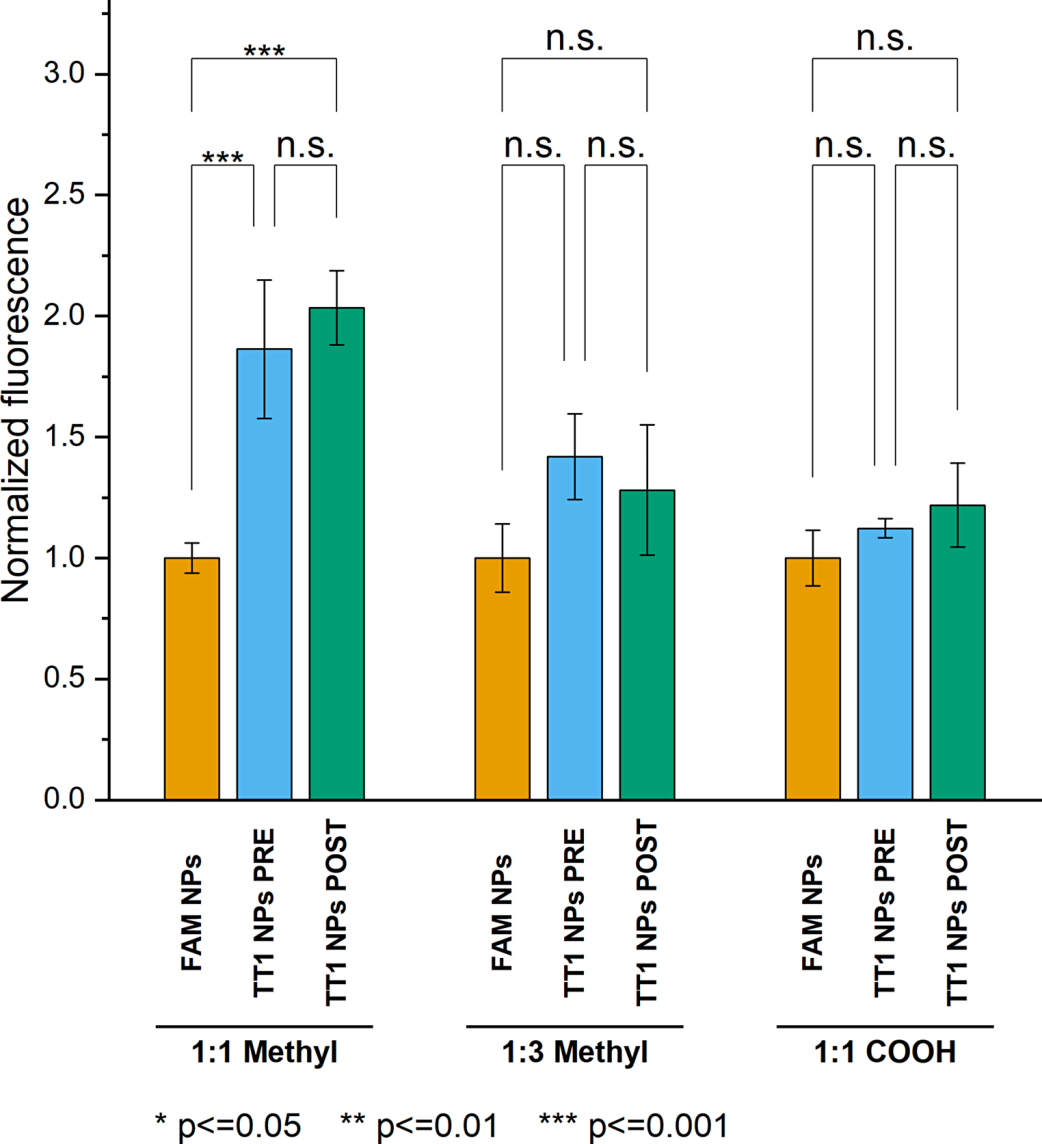
Binding of NPs to Ni-NTA agarose beads coated with 6xHis-tagged p32 protein. Three replicates were performed per sample. Statistical significance was evaluated by pairwise comparison using Tukey’s significance test and significance limit was set at *p* ≤ 0.05

The ability of the NPs to bind to the target protein p32 was evaluated using a cell-free binding assay. The assay is based on agarose beads coated with nickel nitrilotriacetic acid (Ni-NTA) complexes that bind reversibly with hexahistidine-tagged proteins [39]. Normalized fluorescence data showed that significant binding was seen only with PCL-PEG-Me 1:1 diluted peptide conjugated samples (Fig. 8). Binding of PCL-PEG-COOH diluted peptide conjugated NPs was not statistically significant compared to control. Furthermore, the data did not indicate any significant differences between pre- and post-conjugated samples in terms of binding affinity to p32, which suggests that both methods are equally good for achieving proper peptide presentation on the NPs’ surface. However, sensitivity of the assay seemed to be limited by unspecific binding, and the inclusion of surfactants in the assay buffers could be evaluated to reduce background signal in peptide-free samples, and thus, improving the assay sensitivity toward target binding.

### Peptide conjugation leads to a morphological shift

**Fig. 9:**
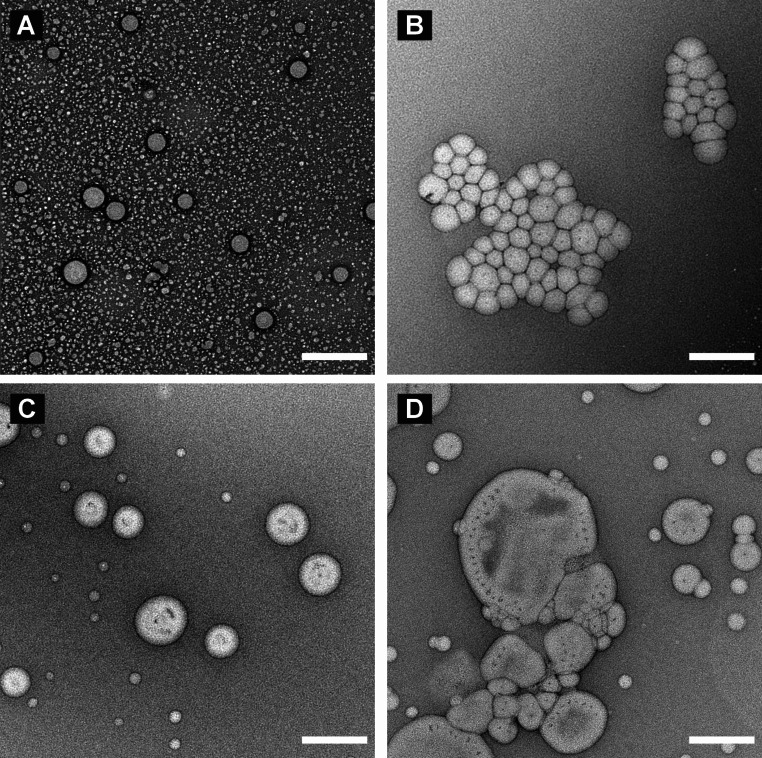
Representative TEM micrographs of non-conjugated and conjugated nanoparticles: (**A**) Non-conjugated NPs (100% PCL-PEG-COOH); (**B**) Blend of PCL-PEG-COOH/FAM (pre-conjugated); (**C**) Blend of PCL-PEG-COOH/TT1 (pre-conjugated); (**D**) Blend of PCL-PEG-COOH/MAL post-conjugated with LinTT1. The samples were stained with uranyl acetate. Scale bars are 200 nm

Negative binding results of PCL-PEG-COOH blended samples prompted us to study further the NP-morphology by transmission electron microscopy (TEM) with negative staining, and differences were observed between the samples (Fig. 9). The NPs prepared from PCL-PEG-COOH base polymer showed a homogeneous population of solid nanospheres with no vesicular shapes (Fig. 9A). Similarly, the NPs prepared from a mixture PCL-PEG-COOH and PCL-PEG-FAM showed a population of solid nanospheres, but mostly in the form of larger agglomerates (Fig. 9B). However, DLS data (Fig. 6) showed that a narrow size distribution (d_H_ 72 nm and PDI 0.12) for this sample, which suggests the agglomerates formed during TEM specimen preparation and are not present in the original dispersion. Peptide-conjugated NPs (Fig. 9C-D) showed a bimodal population of solid nanospheres and vesicles (polymersomes). The vesicular particles are present both in pre-conjugated (Fig. 9C) and post-conjugated (Fig. 9D) and their presence correlated with an increased hydrodynamic size and PDI (Fig. 6). The data suggest that the morphological shift occurs independently of the conjugation strategy (pre vs. post). As discussed above, the post-conjugation approach led to less a homogeneous particle size distribution than the pre-conjugation (Fig. 6), and this was also clearly visible in TEM (Fig. 9C-D).

The peptide conjugation essentially increases the length of the hydrophilic segment of the ABC, which can be expected to change the hydrophilic/lipophilic balance and affect the self-assembly, as the equilibrium morphology if essentially determined by the block length ratio [11]. Furthermore, for this mixture of polymers with cationic and anionic PEG termini, ionic interactions will contribute to the force balance during polymer self-assembly. The observation of what appear to be vesicles with aqueous cores provides further support to the explanation that a subpopulation of the peptide ligands can be trapped inside the particles, causing a reduction in net fluorescence (Fig. 7A).

The use of pre-conjugated polymers for NPs preparation has been established in the literature for various diblock systems, including PEG-PCL [10, 26, 31]. However, in these reports, polymers with longer PEG blocks have been typically used [9, 26]. The colloidal stability challenges and morphological transition seen with the present work can, therefore, be related to a relatively short PEG block and its influence on steric stabilization against agglomeration and on the preferred aqueous morphology. Additional research is required for understand the effect of peptide conjugation on self-assembly and how it is related to the hydrophilic-lipophilic balance of the base polymer.

### Purification of polymer-peptide conjugate by precipitation

Precipitation-based purification of PCL-PEG-TT1 was briefly explored as a means to remove unreacted free peptide and base polymer. Dichloromethane is an excellent solvent for the base polymer, so we first attempted to dissolve the lyophilized reaction product in DCM, and an orange mixture containing precipitate formed. When the mixture was filtered by vacuum through a sintered glass filter, a clear colorless solution passed through, which suggests that the peptide-polymer conjugate was not soluble in DCM. When DCM was evaporated, a colorless polymer film formed, which indicates only the unreacted polymer dissolved in DCM. The presence of unreacted polymer was expected based on the analyzed 62% conversion degree of base polymer to PCL-PEG-MAL. We noticed that the orange precipitate was soluble in acetone, so we attempted to prepare the NPs from the residue by dissolving it in acetone, adding several volumes of water and measuring the NP’s size distribution by DLS. The NPs formed and they were in typical size range of the NPs prepared from PCL-PEG of this molecular weight (d_H_ = 82 nm, PDI 0.18). These findings suggest that the peptide-polymer conjugate was insoluble in DCM and soluble in acetone.

Dry acetone was known to be a poor solvent for the free peptide, and so removal of free peptide from the conjugate by acetone precipitation was attempted in a separate experiment. Dissolution of the sample in pure acetone resulted in the formation a greenish solution with orange precipitate. The precipitate was removed by centrifugation and the solution dried under N_2_ flow, affording a greenish solid film. This film was used to prepare NPs by the nanoprecipitation method, and they showed a ligand concentration of 6.5 nmol/mg and average d_H_ of 110 nm at PDI 0.13. These experiments suggest precipitation in selective organic solvents could be used to purify the polymer-peptide conjugate. However, tests with larger sample amounts with purity and yield calculations would be required to verify the results.

## Conclusions

In this work, we studied the feasibility of a pre-conjugation strategy for the attachment of a short, fluorescently labelled, cationic peptide to PCL-PEG NPs with low hydrophilic block volume fraction $\:\left(f\right)$. We compared the pre- and post-conjugation approaches with regards to the physicochemical properties and target binding ability of the NPs. The results indicate that the conjugation of this peptide to the PEG terminus influenced the self-assembly behavior of PEG-*b*-PCL diblock copolymer, resulting in a morphological transformation from solid nanospheres to vesicles. The morphological shift was independent of the conjugation strategy and was associated with significant increases in particle size. Interestingly, NPs prepared using the pre-conjugation approach showed a more homogeneous particle size distribution, smaller average particle size and higher colloidal stability during post-processing compared to post-conjugated NPs. The observed morphology change was also associated with reduced fluorescence yield per ligand amount in peptide-conjugated NPs compared to controls, which can potentially be explained by the peptide’s entrapment inside the vesicle lumen, leading to fluorescence self-quenching due to high local fluorophore concentration. Significant target protein binding was achieved for PCL-PEG-Me-diluted conjugate, regardless of the applied conjugation strategy. However, binding was dependent on the diluent polymer used.

The use of GPC was explored as a method for the analysis of peptide-polymer conjugates, to detect and quantify the attached ligand and to enable simultaneous molecular weight determination. While the free peptide was easily separated from water-soluble polymers by gel permeation, its separation from a copolymer with a highly hydrophobic PCL block was more challenging due to contradicting solubility requirements. Overall, this case study indicates that the preparation of targeted, low $\:f$ PCL-PEG NPs from pre-synthesized peptide-polymer conjugates can be a feasible approach and the pre-conjugated NPs showed successful binding to their target protein. However, the data shows that the self-assembly of this block copolymer was influenced by peptide conjugation, and these effects should be carefully evaluated when designing and preparing targeted polymer NPs. Further research in this direction should involve the expansion of this study to different block molecular weights and different peptides, for improved understanding of the effects on peptide conjugation the formation of PEG-PCL nanoparticles and how it is related to the hydrophilic-lipophilic balance of the polymer.

## Electronic Supplementary Material

Below is the link to the electronic supplementary material.

Supplementary information

## Data Availability

All relevant data from the study is included in this publication.
