# Supplementary material for: Effects of LinTT1-peptide conjugation on the properties of poly(ethylene glycol)-block-(ε-caprolactone) nanoparticles prepared by the nanoprecipitation method
Source: Drug Deliv Transl Res. 2025 Jan 3;15(8):2733–48. doi: 10.1007/s13346-024-01768-7 (PMC12213848; doi:10.1007/s13346-024-01768-7)
Supplement: Supplementary file 1 — Supplementary information [file 13346_2024_1768_MOESM1_ESM.pdf]

SUPPORTING INFORMATION

Effects of LinTT1-peptide conjugation on the properties of poly(ethylene glycol)-block-(ε-caprolactone) nanoparticles prepared by the nanoprecipitation method

Voitto Känkänen [a,b,*], Sami-Pekka Hirvonen [c], Tambet Teesalu [d,e], Jouni Hirvonen [a], Vimalkumar Balasubramanian [f,*], Hélder A. Santos [a,g,*]

[a] Drug Research Program, Division of Pharmaceutical Chemistry and Technology, Faculty of Pharmacy, University of Helsinki, FI-00014 Helsinki, Finland

[b] Department of Applied Physics, School of Science, Aalto University, FI-02150, Espoo, Finland

[c] Department of Chemistry, Faculty of Science, University of Helsinki, P.O. Box 55, 00014 Helsinki, Finland

[d] Laboratory of Precision- and Nanomedicine, Institute of Biomedicine and Translational Medicine, University of Tartu, Tartu, 50411 Estonia

[e] Materials Research Laboratory, University of California, Santa Barbara, CA 93106, USA.

[f] Chemical and Pharmaceutical Development, Bayer Oy, FI-20210 Turku, Finland

[g] Department of Biomaterials and Biomedical Technology, The Personalized Medicine Research Institute (PRECISION), University Medical Center Groningen, University of Groningen, Ant. Deusinglaan 1, Groningen, 9713 AV, The Netherlands

*Corresponding authors: voitto.kankanen@helsinki.fi (V. Känkänen), vimalkumar.balasubramanian@bayer.com (V. Balasubramanian), h.a.santos@umcg.nl (H.A. Santos)

Colloidal stability evaluation in PBS with and without poloxamer

1. Characterization of polymers

The polymers used in this experiment are listed in **Table S1**. Molecular weights were determined by gel permeation chromatography (GPC) in tetrahydrofuran against polystyrene standards. The GPC data in Table S1 indicates that there is a large difference in molecular weight between the PCL-PEG-Me and PCL-PEG-MAL, even though the nominal block lengths reported by the suppliers were similar.

Table S1. Molecular weight analysis results for the polymers used in this stabilization experiment.

				**GPC results**		
**Type**	**PEG terminus**	**Nominal PEG M_n (g/mol)**	**Nominal PCL M_n (g/mol)**	**M_n (g/mol)**	**M_w (g/mol)**	**Đ**
PCL-PEG-MAL	Maleimide	2,000	10,000	14,864	34,312	2.31
PCL-PEG-Me	Methyl ether	2,000	6,000	11,398	12,786	1.12
PCL-PEG-Me	Methyl ether	2,000	10,000	12,104	14,573	1.20

2. Preparation of NPs

NPs were prepared using the batch nanoprecipitation method was used. Polymers were dissolved in acetone at 10 mg/mL and 3.0 mL of the solution added dropwise to 15.0 mL of aqueous phase under magnetic stirring. Particles were prepared from PCL-PEG-Me and from a blend of PCL-PEG-MAL and PCL-PEG-Me, as shown below.

Polymer solution 1: 5.0 mg/mL PCL-PEG-MAL + 5.0 mg/mL PCL-PEG-Me 2k-10k
Polymer solution 2: 10 mg/mL PCL-PEG-Me 2k-10k
Polymer solution 3: 10 mg/mL PCL-PEG-Me 2k-6k

Two different aqueous phases were used, to see the inclusion of surfactant from the very beginning would result in further improvement of stability.

Aqueous phase 1: Milli-Q water

Aqueous phase 2: Milli-Q water with 1 % P188 w/v

Each batch of particles (30 mg) was divided into two parts for centrifugal ultrafiltration testing. Centrifugal ultrafiltration was performed using Microsep Advance filtration devices (PALL) with 100k MWCO Omega (PES, polyether sulfone) membrane. The filter devices were rinsed with Milli-Q water before use. A centrifuge with temperature control was used and the temperature was adjusted to 21 °C.

The following buffers were used for the buffer exchange and purification by ultrafiltration.

Buffer 1: 1xPBS

Buffer 2: 1xPBS with 1 % P188 w/v

The prepared samples (15 mg NPs) were transferred to the rinsed filtration tubes and diluted to 15 mL with the buffer. Then, filtration at 2500 rcf was continued until the retentate was at 2 – 3 mL, corresponding to 5–7.5 mg/mL NP concentration. The dilution-concentration cycle was repeated three times for each sample. The times required to achieve 2–3 mL retentate volume are shown in the **Table S2**. Centrifugation times were relatively long for a buffer exchange process, as up to 3 hours per cycle were required for some samples.

Table S2. Centrifugation times required to reach 2-3 mL retentate volume.

<table>
<tr><th>Polymer solution</th><th>Nanoprecip. Aqueous phase</th><th>Ultrafiltration buffer</th><th>Filtration cycle</th><th>Centrifugation time required (min)</th></tr>
<tr><td rowspan="6">1</td><td rowspan="3">1</td><td rowspan="3">PBS</td><td>1</td><td>14 min @ 1000 rcf
+ 25 min @ 2000 rcf
+ 30 min @ 2500 rcf</td></tr>
<tr><td>2</td><td>66 min @ 2500 rcf</td></tr>
<tr><td>3</td><td>83 min @ 2500 rcf</td></tr>
<tr><td rowspan="3">1</td><td rowspan="3">PBS + 1% P188</td><td>1</td><td>14 min @ 1000 rcf
+ 25 min @ 2000 rcf
+ 30 min @ 2500 rcf</td></tr>
<tr><td>2</td><td>72 min @ 2500 rcf</td></tr>
<tr><td>3</td><td>108 min @ 2500 rcf</td></tr>
</table>

Polymer solution	Nanoprecip. Aqueous phase	Ultrafiltration buffer	Filtration cycle	Centrifugation time required (min)
	2	PBS	1	14 min @ 1000 rcf<br>+ 25 min @ 2000 rcf<br>+ 30 min @ 2500 rcf
			2	60 min @ 2500 rcf
			3	83 min @ 2500 rcf
	2	PBS + 1% P188	1	14 min @ 1000 rcf<br>+ 25 min @ 2000 rcf<br>+ 30 min @ 2500 rcf
			2	72 min @ 2500 rcf
			3	108 min @ 2500 rcf
2	1	PBS	1	104 min @ 2500 rcf
			2	169 min @ 2500 rcf
			3	195 min @2500 rcf
	1	PBS + 1% P188	1	104 min @ 2500 rcf
			2	154 min @ 2500 rcf
			3	195 min @2500 rcf
3	1	PBS	1	77 min @ 2500 rcf
			2	109 min @ 2500 rcf
			3	95 min @ 2500 rcf
	1	PBS + 1% P188	1	55 min @ 2500 rcf
			2	109 min @ 2500 rcf
			3	115 min @ 2500 rcf

The samples were inspected visually by naked eye before and after the buffer exchange process. The results are shown in **Table S3** and **Figures S1**–**S2**. For the sample containing PCL-PEG-MAL, the addition of P188 was not able to fully stabilize the dispersion, even though the presence of large agglomerates was reduced by addition of the surfactant. For the PCL-PEG-Me NPs, addition of P188 to PBS buffer prevented the formation of visible agglomerates during centrifugal ultrafiltration and agitation in a vial.

Table S3. results of visual inspection of NP samples before and after buffer exhange by centrifugal ultrafiltration.

Polymer blend	Nanoprecip. Aqueous phase	Ultrafiltration buffer	Before buffer exchange	After buffer exchange
1	1	PBS	Almost clear, no visible particles	Opaque, many visible fiber-like particles
	1	PBS + 1% P188	Slightly opaque, a few visible particles	Opaque, many visible fiber-like particles
	2	PBS	Almost clear, no visible particles	Opaque, some large particles, vortical patterns appear upon mixing
	2	PBS + 1% P188	Slightly opaque, a few visible particles	Opaque, some large particles, vortical patterns appear upon mixing
2	1	PBS	Clear, no visible particles	Clear, many fiber-like large particles
	1	PBS + 1% P188	Clear, no visible particles	Clear, no visible particles
3	1	PBS	Clear, no visible particles	Clear, few large particles
	1	PBS + 1% P188	Clear, no visible particles	Clear, no visible particles

Supplementary Figures

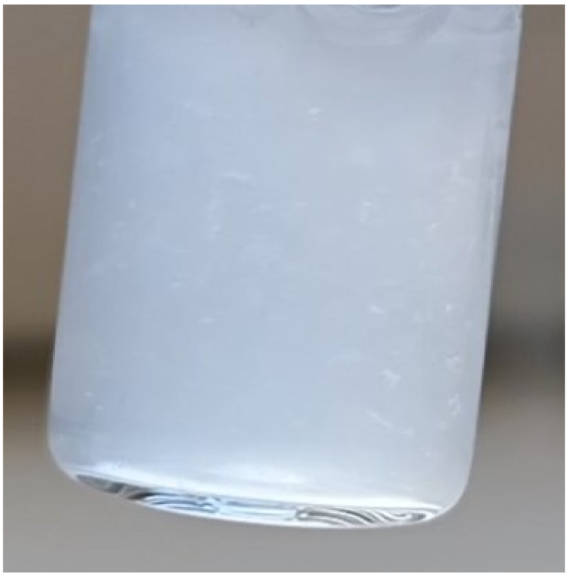

a) PCL-PEG-MAL/PCL-PEG-Me NPs, prepared in water, buffer exchanged to PBS.

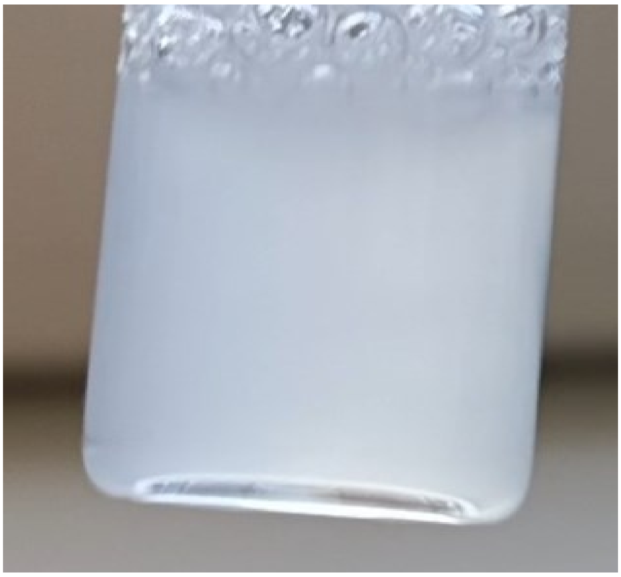

b) PCL-PEG-MAL/PCL-PEG-Me NPs, prepared in water, buffer exchanged to PBS + 1% P188.

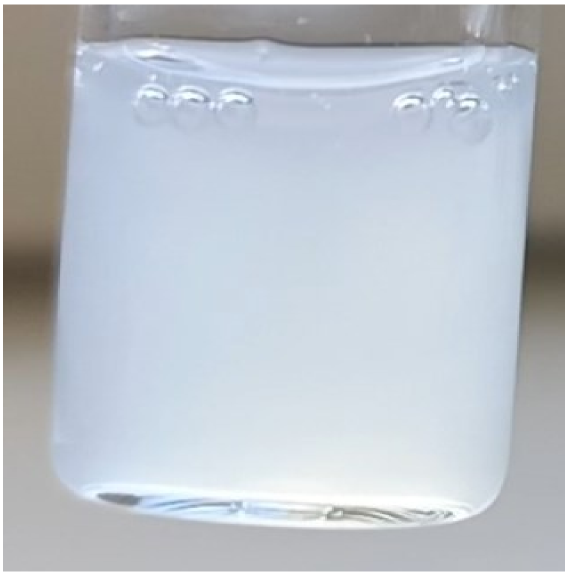

c) PCL-PEG-MAL/PCL-PEG-Me NPs, prepared in 1% P188, buffer exchanged to PBS.

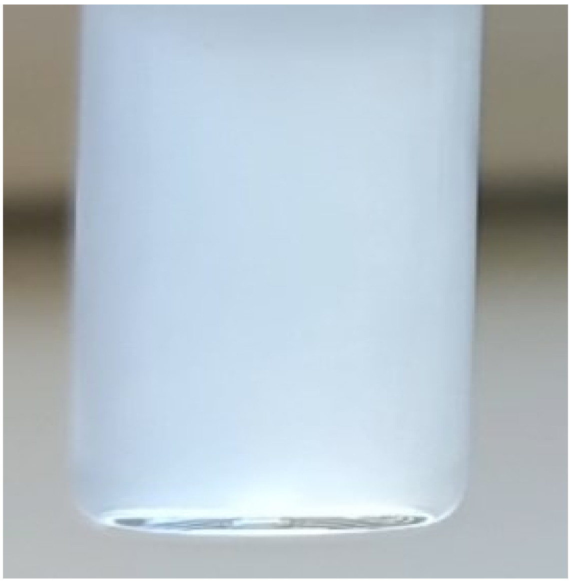

d) PCL-PEG-MAL/PCL-PEG-Me NPs, prepared in 1% P188, buffer exchanged to PBS + 1% P188.

Figure S1. (a–d) Example photographs taken from samples upon agitation of PCL-PEG-Me/MAL blend NPs collected from the centrifugal ultrafiltration tube filters after buffer exchange. The samples were agitated strongly by hand.

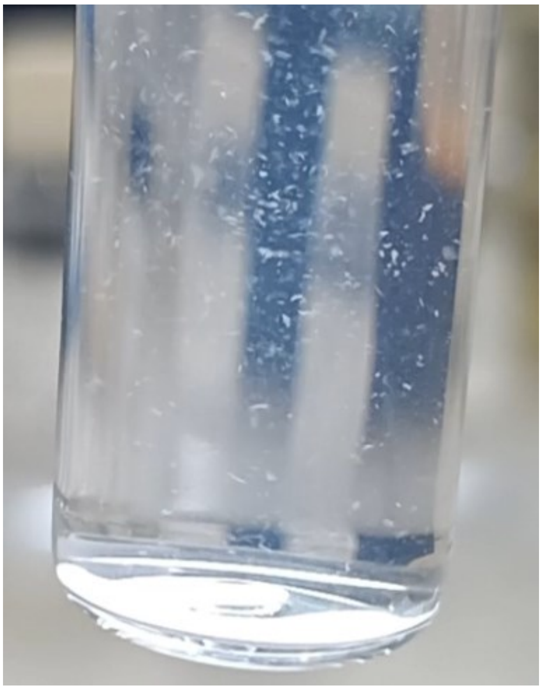

a) PCL-PEG-Me 10k-2k NPs, buffer exchanged to PBS.

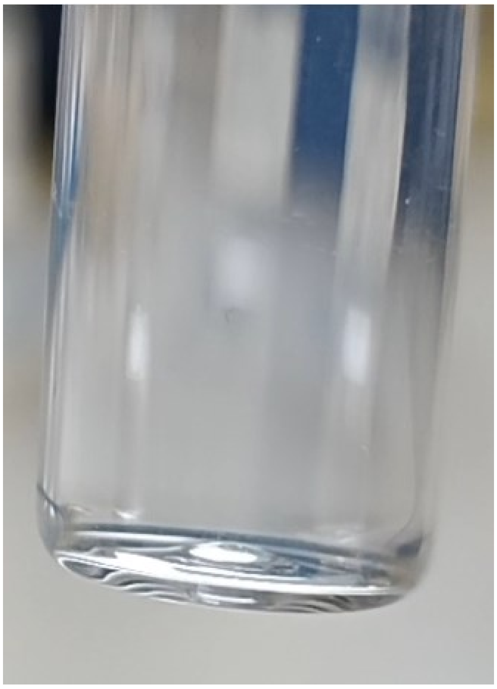

b) PCL-PEG-Me 10k-2k NPs, buffer exchanged to PBS + 1% P188.

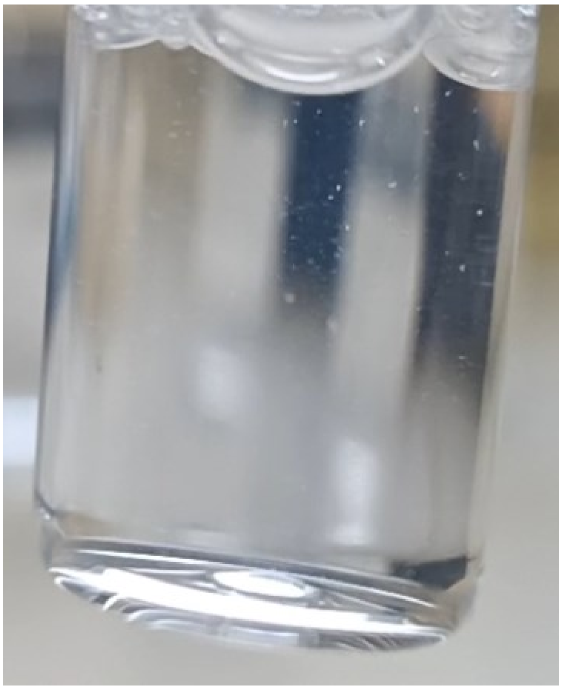

c) PCL-PEG-Me 6k-2k NPs, buffer exchanged to PBS.

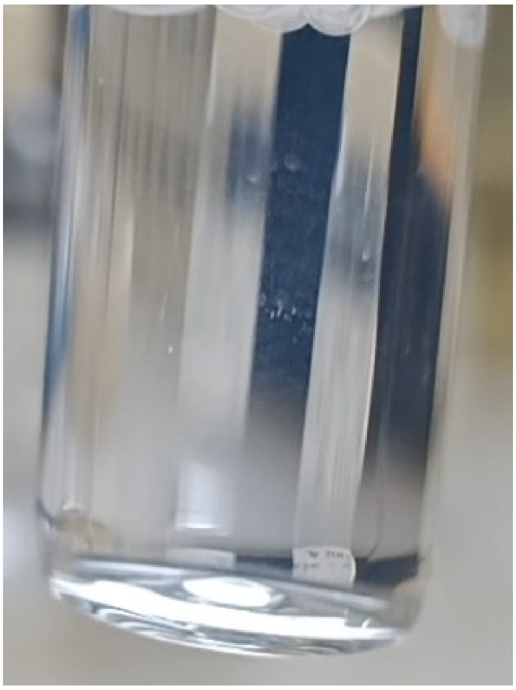

d) PCL-PEG-Me 6k-2k NPs, buffer exchanged to PBS + 1 % P188.

Figure S2. (a–d) Photographs of PCL-PEG-Me NP samples collected from the centrifugal ultrafiltration tube filters after buffer exchange. The samples were agitated strongly by hand.

GPC control experiments in aqueous mobile phase

Control experiments were performed on the amine-functionalized acrylate copolymer column (NOVEMA Max) to understand the elution behavior of water-soluble sample components. In the control experiments, 0.1 M $NaNO_3$ + 3% ACN (HPLC grade, Sigma-Aldrich) in ultrapure water, filtered through 0.45 µm PTFE filters (PALL), was used as the mobile phase. Flow rate 0.800 mL/min and column temperature + 30 °C were used. Samples were dissolved in eluent and filtered through Clarify 0.22 um PTFE filters.

It was not known if the anionic nature of carboxylic acid terminated PCL-PEG-COOH could cause ionic interactions with the column intended for the analysis of cationic polymers. The possible effect of PEG terminal group on elution was tested by analyzing hydroxyl- and acid-terminated PEGs on the NOVEMA Max column. A PEG narrow molecular weight standard (PEG-OH, M_p = 982 g/mol) was obtained from Polymer Standard Service and it was assumed to be hydroxyl terminated. Methoxypolyethylene glygol 1,000 propionic acid (Me-PEG-COOH, M_n = 1000 g/mol) was obtained from Sigma Aldrich. The chromatograms (**Figure S3**) showed nearly identical elution of the two control samples, indicating that the elution in this mobile phase is based on size and not confounded by ionic interactions.

Separation of Cys-FAM-Ahx-AKRGARSTA-OH from poloxamer was also tested as a control. A 1.00 mg/mL solution of peptide in 1×PBS pH 7.4 + 0.25 % P188 was diluted 1:11 with the eluent and filtered prior to injection. The chromatograms (**Figure S4**) show elution of three main peaks in RI channel and two peaks in UV channel (400 nm). The peak at 9.5 mL was attributed to poloxamer 188 and peaks at 10.8 mL and 11.5 mL were attributed to dimers and monomers of the peptide, respectively. Corresponding peaks related to FAM are visible in the UV channel. Comparison to peak position of the M_p 982 g/mol PEG standard shows that the peptide assumed a significantly smaller hydrodynamic volume per molecular weight compared to PEG in this mobile phase (**Figure S5**).

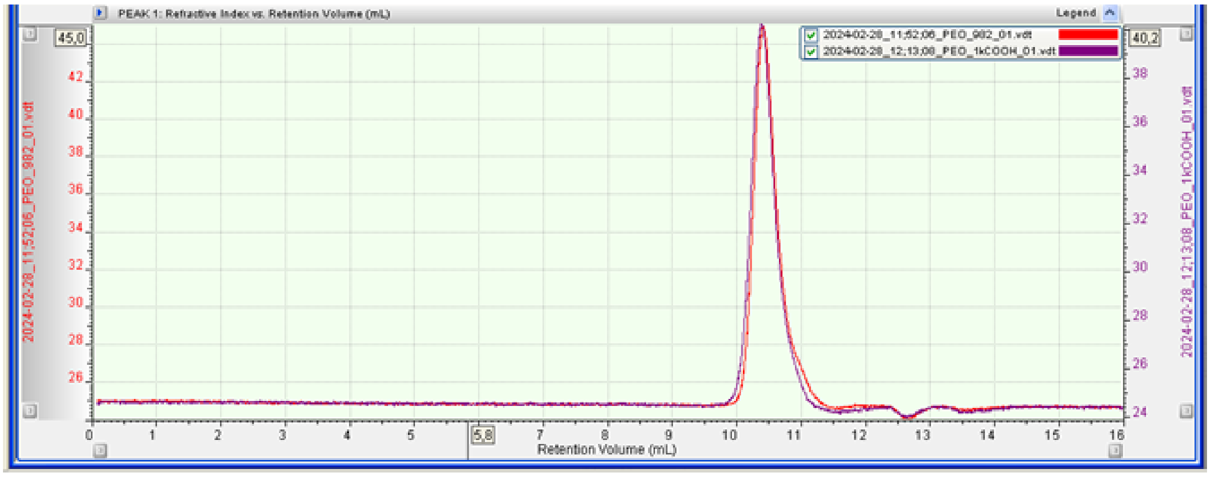

Figure S3. Terminal carboxylic acid group did not influence elution volume of PEG. Samples PEG-OH M_p 982 g/mol (red) and PEG-COOH M_n 1,000 g/mol (purple). Refractive index signal is shown in red and UV channel at 400 nm in purple. The sample was run in 0.10 M NaNO3 + 3 % ACN (aq.) mobile phase on a NOVEMA Max column.

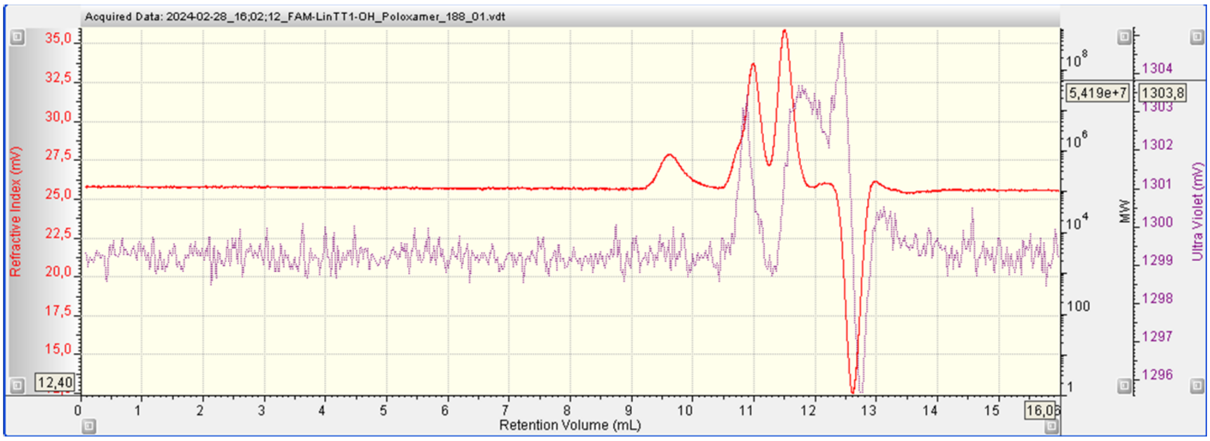

Figure S4. GPC chromatogram of peptide solution in PBS pH 7.4 + 0.25 % Poloxamer 188. The sample was run in 0.10 M $NaNO_3$ + 3 % ACN (aq.) mobile phase on a NOVEMA Max column.

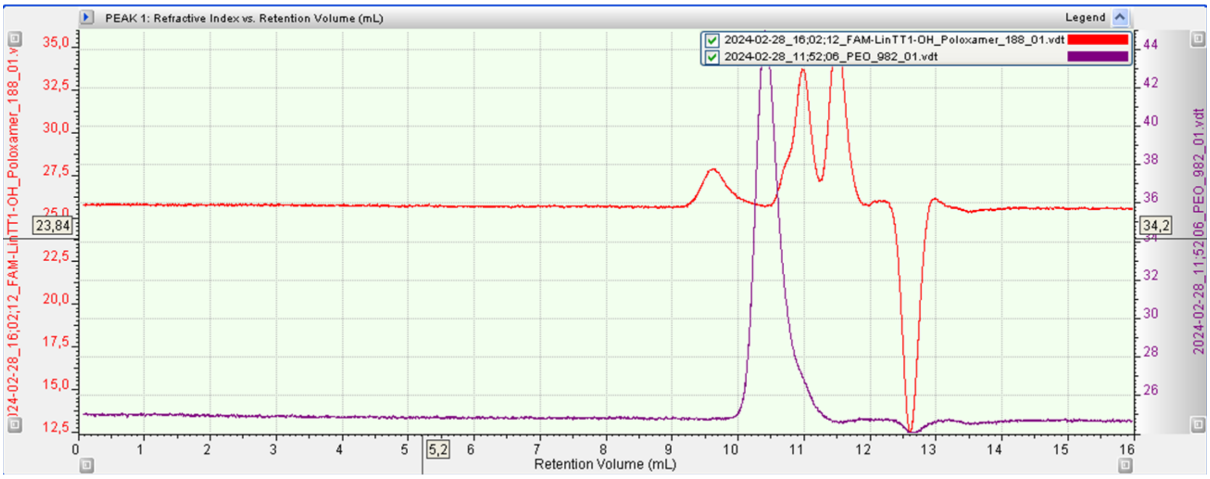

Figure S5. GPC chromatogram of peptide in PBS pH 7.4 + 0.25 % Poloxamer 188 (red, above) and PEO standard 982 g/mol (purple, below). The samples were run in 0.10 M $NaNO_3$ + 3 % ACN (aq.) mobile phase on a NOVEMA Max column. Only refractive index channel data is shown.
